# Extracellular vesicles as a source of prostate cancer biomarkers in liquid biopsies: a decade of research

**DOI:** 10.1038/s41416-021-01610-8

**Published:** 2021-11-22

**Authors:** Manuel Ramirez-Garrastacho, Cristina Bajo-Santos, Aija Line, Elena S. Martens-Uzunova, Jesus Martinez de la Fuente, Maria Moros, Carolina Soekmadji, Kristin Austlid Tasken, Alicia Llorente

**Affiliations:** 1 Department of Molecular Cell Biology, Institute for Cancer Research, Oslo University Hospital, Oslo, Norway; 2 Latvian Biomedical Research and Study Centre, Riga, Latvia; 3 Erasmus MC Cancer Institute, University Medical Center Rotterdam, Department of Urology, Laboratory of Experimental Urology, Erasmus MC, Rotterdam, The Netherlands; 4 Instituto de Nanociencia y Materiales de Aragón (INMA), CSIC-Universidad de Zaragoza, Zaragoza, Spain; 5 Biomedical Research Networking Center in Bioengineering, Biomaterials and Nanomedicine (CIBER-BBN), Madrid, Spain; 6 Department of Cell and Molecular Biology, QIMR Berghofer Medical Research Institute, Brisbane, QLD Australia; 7 School of Biomedical Sciences, Faculty of Medicine, University of Queensland, Brisbane, QLD Australia; 8 Department of Tumor Biology, Institute for Cancer Research, Oslo University Hospital, Oslo, Norway; 9 Institute of Clinical Medicine, University of Oslo, Oslo, Norway; 10 Department for Mechanical, Electronics and Chemical Engineering, Oslo Metropolitan University, Oslo, Norway

**Keywords:** Prostate cancer, Biomarkers

## Abstract

Prostate cancer is a global cancer burden and considerable effort has been made through the years to identify biomarkers for the disease. Approximately a decade ago, the potential of analysing extracellular vesicles in liquid biopsies started to be envisaged. This was the beginning of a new exciting area of research investigating the rich molecular treasure found in extracellular vesicles to identify biomarkers for a variety of diseases. Vesicles released from prostate cancer cells and cells of the tumour microenvironment carry molecular information about the disease that can be analysed in several biological fluids. Numerous studies document the interest of researchers in this field of research. However, methodological issues such as the isolation of vesicles have been challenging. Remarkably, novel technologies, including those based on nanotechnology, show promise for the further development and clinical use of extracellular vesicles as liquid biomarkers. Development of biomarkers is a long and complicated process, and there are still not many biomarkers based on extracellular vesicles in clinical use. However, the knowledge acquired during the last decade constitutes a solid basis for the future development of liquid biopsy tests for prostate cancer. These are urgently needed to bring prostate cancer treatment to the next level in precision medicine.

## Background

### Prostate cancer

In 2020, almost 20 million people were diagnosed with cancer and 10 million were estimated to die of cancer worldwide [1]. Prostate cancer (PCa) was the most frequent cancer type among men in 112 countries and the second leading cause of cancer deaths. It is expected that improving the diagnosis and treatment of PCa patients will increase men’s life expectancy.

Prostate cancer is classified as localised, locally advanced or metastatic disease. Localised PCa is further subdivided into risk groups based on prostate-specific antigen (PSA) level, International Society of Urological Pathology (ISUP) grade/Gleason score (GS) and clinical TNM stage [2, 3]. In general, low-risk patients are offered active surveillance (AS) and intermediate-risk patients are treated by radical prostatectomy (RP) or curative radiotherapy (RT). High-risk patients are treated with RP with extended lymph-node dissection or RT in combination with long-term androgen-deprivation therapy. Locally advanced patients are offered extended lymph-node dissection and RP or RT as part of multimodal therapy. Metastatic disease is at present incurable, and these patients are offered systemic treatment, eventually in combination with surgery or RT.

The incidence of PCa increased dramatically when PSA testing for early detection and screening of PCa was introduced into the market in the 1990s [4, 5]. Overdiagnosis and subsequent overtreatment became a problem, and the search for biomarkers  that could discriminate indolent localised PCa that can be followed by AS from aggressive localised PCa that needs radical treatment was intensified. Thirty years later, a handful of molecular biomarkers are finally slowly approaching the clinic, such as the prostate cancer antigen 3 (PCA3) RNA test or the SelectMDx test based on RNA detection of DLX1 and HOXC6, both using urine collected after prostate massage [4, 6–8]. These tests can improve detection of clinically significant PCa and change clinical decisions for patients within each risk group. They are, however, still not routinely recommended in the clinical guidelines as more data are needed to prove their cost–benefit. At the same time, the treatment landscape of metastatic PCa is rapidly changing [9]. As new expensive drugs are entering the clinic, there is an intense search for predictive biomarkers that aim to identify responsive patients and thereby reduce unnecessary side effects.

PCa is a multifocal and heterogeneous malignancy. To bring precision medicine in PCa treatment to the next level, we need to identify biomarkers reflecting the phenotype of multiple tumour foci, which is determined by the cancer-cell genotype and shaped by the tumour microenvironment and systemic factors. The use of liquid biopsies constitutes an attractive approach in this respect because the intratumoural heterogeneity within and between the tumour foci can potentially be mirrored by molecular analyses of body fluids. Body fluids are easily accessible, enabling screening of men at risk of developing PCa as well as real-time monitoring of disease progression and treatment responses. In fact, molecular biomarkers in liquid biopsies have a long history in PCa. This is exemplified by the use of prostatic acid phosphatase (PAP) for the diagnosis of PCa since 1938 [10] and later PSA, which was FDA-approved to monitor PCa relapse in 1986 [5].

### Liquid biopsies

Liquid biopsies have emerged as a promising alternative to tissue biopsies for the detection, prognosis and prediction of response to therapy, AS and post-operative monitoring of PCa. The term ‘liquid biopsies’ refers to the analysis of tumour cells and molecules providing information about the disease in samples of body fluids like blood or urine [11]. Such samples can be obtained in a minimally invasive or non-invasive way; therefore, liquid biopsies are particularly suitable for monitoring patients and tracking tumour evolution. Commonly studied cancer-derived analytes in liquid biopsies are circulating tumour cells (CTCs) and circulating tumour DNA (ctDNA) [12, 13]. CTCs are disseminated cancer cells that may exist in the circulation as single cells or clusters of 2–50 cells consisting of only CTCs or CTCs associated with stromal or immune cells [14]. The methods for CTC analyses range from enumeration of CTCs, which can be exploited for prognosis and early detection of relapse, to genomic, transcriptomic and proteomic profiling of CTCs and establishing ex vivo cultures or xenografts that may be of use for guiding the choice of drug treatment [15]. However, the main challenges in CTC clinical use are their very low counts in peripheral blood and their phenotypic heterogeneity [16, 17]. In localised PCa, CTCs are detectable only in a minority of patients [18, 19]. However, CTCs are detectable in 33–75% of patients with metastatic castration-resistant prostate cancer (mCRPC) and have a high prognostic and predictive significance [20–23].

Cell-free DNA (cfDNA) fragments are released into the circulation from a variety of cell types. Tumour cell-free DNA (ctDNA) represents a fraction of cfDNA that is released from apoptotic or necrotic tumour cells. ctDNA can be distinguished from normal tissue-derived cfDNA by the presence of genetic or epigenetic alterations such as somatic point mutations, rearrangements, copy number variations and tumour-specific methylation markers [17]. The half-life of ctDNA varies from around 16 min to 2.5 h, allowing real-time monitoring of tumour burden [24, 25]. Hence, ctDNA analyses could be applied for monitoring treatment responses and disease progression, and tracking intratumoural heterogeneity and evolution [26]. However, the fraction of ctDNA in the cfDNA may vary from 0.01 to 90%, and ultrasensitive methods such as digital-droplet PCR, BEAMing or tagged amplicon sequencing are required for the detection of rare tumour-derived variants in the background of wild-type cfDNA [27]. Another challenge in the clinical application of ctDNA assays is that a fraction of the genetic alterations in the cfDNA may arise from age-related clonal expansion of mutated hematopoietic cells [28].

### Extracellular vesicles

EVs represent an alternative source of cancer-derived molecules in liquid biopsies [16, 17, 29, 30]. ‘EV’ is a generic term for all types of lipid bilayer-delimited particles that are naturally released from cells and cannot replicate [31]. According to the biogenesis pathway, the main subtypes of EVs are exosomes, microvesicles (also called ectosomes, shedding vesicles or microparticles) and apoptotic bodies [32–35]. Exosomes correspond to the released intraluminal vesicles found in the lumen of multivesicular bodies and range in size from 30 to 150 nm. Microvesicles are formed by budding and blebbing from the plasma membrane and the majority have a size range from 100 to 1000 nm [36]. Finally, apoptotic bodies are formed by blebbing of the plasma membrane or formation of membrane protrusions such as microtubule spikes, apoptopodia and beaded apoptopodia in apoptotic cells. The majority of apoptotic bodies range in size from 1 to 5 µm in diameter, though the formation of smaller vesicles during the progression of apoptosis has also been reported [37]. In PCa, large EVs (1–10 μm), usually referred to as large oncosomes, have been found to be released by shedding of membrane blebs from highly migratory cancer cells, but their biogenesis is not fully understood [38, 39]. Although the mean size of various EV subtypes is different, their size range overlaps and the current EV-isolation methods do not allow accurate separation of the EV subtypes. Therefore, the International Society for Extracellular Vesicles recommends using operational terms for EV subtypes referring to their physical or biochemical characteristics instead of the terms ‘exosome’ or 'microvesicle', unless their biogenesis pathway is clearly established [31].

EVs are secreted by virtually all cell types in the body and are able to reach various body fluids, including blood, urine, semen, milk, saliva, etc. [32, 40, 41]. There is not much known about the specific mechanism of EV release into body fluids, and vesicles formed by different mechanisms and cell types are expected to coexist in biofluids. Thus, vesicles that are found in biofluids would be more appropriately referred to as EVs. This is the term that will be used in this review, even if other terms may have been used in the original articles.

Although initially considered to be a waste-disposal mechanism [42], it is now clear that both EVs generated by living or apoptotic cells can be taken up by recipient cells and are important mediators of intercellular communication [37, 43]. A growing body of evidence suggests that cancer-derived EVs promote cancer progression by acting in a paracrine and systemic manner: they transfer aggressive phenotypic features and drug resistance to other cells, mediate the cross-talk with stromal cells and bone marrow, modulate the antitumour immune response and promote the formation of pre-metastatic niches [30, 44, 45].

EVs carry a variety of proteins, lipids, carbohydrates (attached to proteins and lipids), coding and non-coding RNAs, DNA fragments, metabolites and even entire organelles, such as in apoptotic bodies and possibly other EV types [32, 46–51]. Their molecular cargo partially reflects the intracellular status and physiological state of their parental cells. EVs isolated from cancer patients’ body fluids have been shown to contain cancer-derived molecules such as truncated EGFRvIII [52], overexpressed MET [53], cancer-specific miRNAs and protein signatures and mutated DNA or mRNA fragments [23, 54–56]. These findings have raised the idea that the analysis of EV molecular cargo could inform about the presence and behaviour of cancer and, therefore, could be of use for diagnosis, monitoring of response to therapy, early detection of relapse and tracking tumour evolution. In fact, emerging evidence shows that DNA molecules in blood-derived EVs show superiority over ctDNA as a cancer biomarker [57, 58]. The study of EVs is a very active area of research at the moment, and several resources have been made available in the last few years to facilitate research in this exciting field (Table 1) [59].

**Table 1:** Some resources for EV research.

Type	Name	Purpose/Description	Web address
EV molecular databases	Exocarta/Vesiclepedia	Compendium of molecular data (protein, RNA and lipid) of EVs from multiple sources.	http://www.exocarta.org/ http://www.microvesicles.org/
	EVpedia	Integrated database of high-throughput molecular data (protein, RNA and lipid) for analyses of prokaryotic and eukaryotic EVs.	http://www.evpedia.info
	exoRBase	Repository of EVs long RNAs (mRNA, lncRNA, and circRNA) derived from RNA-seq data analyses in different human body fluids.	http://www.exoRBase.org
	exRNA Atlas	Data repository of the Extracellular RNA Communication Consortium including small RNA sequencing and qPCR-derived exRNA profiles from human and mouse biofluids.	https://www.exrna-atlas.org/
Courses	Basics of Extracellular Vesicles	This MOOC course provides basic knowledge about EVs.	https://www.coursera.org/learn/extracellular-vesicles
	Extracellular Vesicles in Health and Disease	This MOOC course provides current understanding about EVs and their role in health and diseases.	https://www.coursera.org/learn/extracellular-vesicles-health-disease
	Extracellular Vesicles: From Biology to Biomedical Applications	Practical course organised by EMBO covering different EV purification and characterisation techniques and strategies to understand the role of EVs in biomedical applications.	https://www.embl.org/about/info/course-and-conference-office/events/exo22-01/
Reporting	EV-TRACK platform	Platform for recording experimental parameters of EV-related studies.	https://www.evtrack.org/
	MIFlowCyt-EV	Framework for standardised reporting of EV flow cytometry experiments.	https://www.tandfonline.com/doi/full/10.1080/20013078.2020.1713526
Guidelines/Position papers	MISEV2018	Provide guidance in standardisation of protocols and reporting in the EV field.	https://www.pubmed.ncbi.nlm.nih.gov/30637094/
	Urinary EVs	A position paper by the Urine Task Force of the International Society for Extracellular Vesicles.	https://www.onlinelibrary.wiley.com/doi/10.1002/jev2.12093
	Blood EVs	Considerations towards a roadmap for collection, handling and storage of blood EVs.	https://www.tandfonline.com/doi/full/10.1080/20013078.2019.1647027
	EV RNA	Obstacles and opportunities in the functional analysis of extracellular vesicle RNA – an ISEV position paper.	https://www.tandfonline.com/doi/full/10.1080/20013078.2017.1286095
	EVs in therapy	Applying EV-based therapeutics in clinical trials – an ISEV position paper.	https://www.tandfonline.com/doi/full/10.3402/jev.v4.30087
Societies /Task Forces/Working groups	ISEV	Global society of EV researchers.	https://www.isev.org/
	National societies	Societies of national EV researchers.	https://www.isev.org/national-societies
	ISEV task forces	The Rigor & Standardization Subcommittee includes several task forces for advancing specific EV areas of research such as urine EVs, blood EVs and reference materials.	https://www.isev.org/rigor-standardization
	EV Flow Cytometry Working Group	This groups aims to establish guidelines for best practices for flow cytometry analysis of EVs.	http://www.evflowcytometry.org
Conferences/Seminars	ISEV Annual Meeting	This seminar brings together EV interested scientists from around the world.	https://www.isev.org/isev-annual-meeting
	WebEVTalk	These online weekly seminars aim to support networking and to push EV science forward.	https://www.youtube.com/user/MsOlinolin/featured
	EV Club	These online weekly seminars are a venue for discussing research and published articles.	https://www.isev.org/ev-club
	Exosomes, Microvesicles and Other Extracellular Vesicles	Keystone symposia are a series of seminars organised for the advancement of biomedical and life sciences.	https://www.keystonesymposia.org/KS/Online/Events/2022B3/Exosomes-Microvesicles-and-Extracellular-Vesicles.aspx?EventKey=2022B3
	Extracellular vesicles	Gordon Research Conferences are a series of seminars bringing a global network of scientists together to discuss frontier research.	https://www.grc.org/extracellular-vesicles-conference/2022/
SpecializedJournals	Journal of extracellular vesicles	Publication of EV research.	https://www.onlinelibrary.wiley.com/journal/20013078
	The European journal of extracellular vesicles	Publication of EV research.	http://www.libpubmedia.co.uk/ejev/
	Extracellular Vesicles and Circulating Nucleic Acids	Publication of EV research.	https://www.evcna.com/
	Journal of extracellular biology	Publication of EV research. (Launching Late 2021)	https://www.isev.memberclicks.net/journal-of-extracellular-biology

*circRNA* circular RNA, *exRNA* extracellular RNA, *lncRNA* long non-coding RNA, *MISEV* minimal information for studies of extracellular vesicles, *ISEV* International Society for Extracellular Vesicles, *MOOC* massive open online course.

**Fig. 1:**
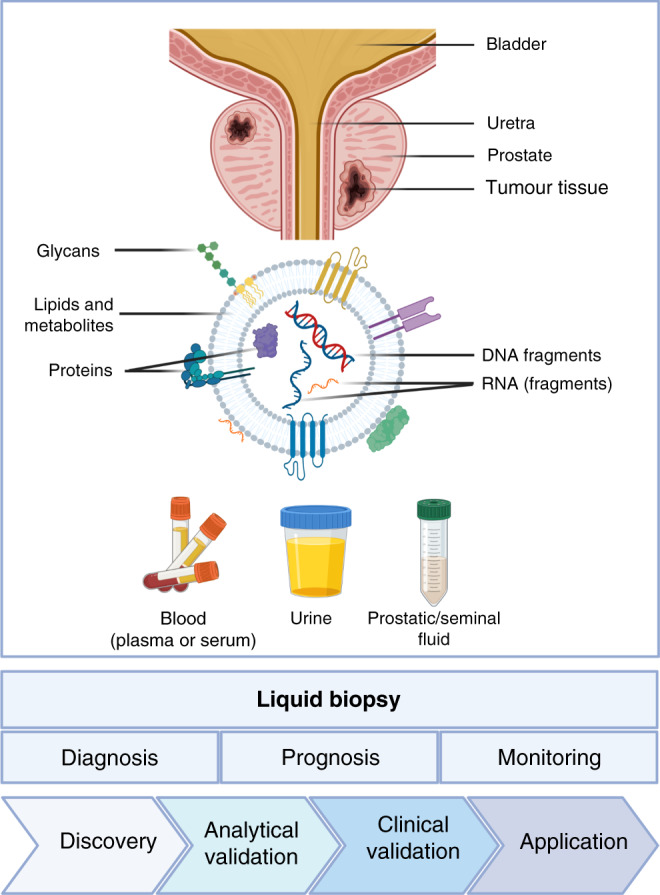
Extracellular vesicles as liquid biopsies for prostate cancer. Figure designed by Elena S. Martens-Uzunova using BioRender.

EV-based biomarkers for PCa have been a very active research area in the last decade [60–69], and the first works already appeared in 2009 [70, 71]. In this review, we discuss the preanalytical and methodological considerations in developing EV-based assays for the diagnosis and management of PCa, and summarise patient studies investigating EV-based biomarkers for diagnosis, prognosis and monitoring of PCa (Fig. 1).

## EVs as liquid biomarkers for prostate cancer: methodological considerations

### Relevant biofluids for the identification of EV-based biomarkers for prostate cancer

Several biofluids are expected to contain prostate-derived EVs [72]. The prostate is an excretory gland of the male genitourinary system located below the bladder, surrounding the proximal urethra, composed of stroma and an epithelium component [73]. The prostatic acinar epithelial cells secrete prostatic fluid, which constitutes approximately one-fifth to one-third of the semen volume and plays an essential role in male fertility [73]. Remarkably, an EV population, called prostasomes, was identified ~40 years ago in prostatic and seminal fluid [74–77]. The highest concentration of prostate-derived EVs can be expected to be found in prostatic fluid and seminal plasma. However, direct collection of prostatic fluid can be relatively invasive and the use of semen for diagnostic purposes of aging PCa patients does not appear as the best option [78]. It should also be mentioned that in addition to the prostate, EVs in seminal fluid may have other origins, such as the epididymis [79]. Importantly, gentle prostate massage can induce the secretion of prostatic fluid into the urethra, which is then mixed with urine during urination. Since prostate massage is often done in connection with a digital rectal examination (DRE), this urine is often called DRE urine. Prostatic fluid is also drained during urination in normal conditions, and possible mechanisms have been proposed [80]. Further, it has been demonstrated that the fraction of prostate-derived EV in urine is significantly enriched after DRE due to the increased amount of prostatic fluid released in the urine [71, 81, 82]. Thus, it could be beneficial to collect urine for EV analysis after DRE to enhance sensitivity. On the other hand, collection of non-DRE urine is more amenable. In any case, urine is seen as a highly suitable and desirable biofluid for liquid biopsy that can be utilised for the clinical management of PCa. Several factors contribute to this, including the minimally invasive character of urine collection, the possibility to collect relatively large volumes and the limited number of organs, i.e., the kidneys, ureters, bladder, seminal vesicles and the prostate (although several recent reports also suggest that EVs from the bloodstream can be found in urine) from which the majority of urinary EVs originate [83]. At the same time, urine is a highly dynamic biofluid and its composition and concentration depend on biorhythm, fitness and diet. This causes a large inter- and intrapersonal variability, which complicates the study of urinary EVs and the discovery and validation of urinary biomarkers in general. Other exogenic factors, such as the presence of microorganism-derived EVs from bacteria and yeast present in urine, as well as viruses with size similar to that of EVs, can additionally contribute to the complexity of the urinary EV population and complicate EV analysis in urine, for example EV quantification [84–90]. The extent by which different organs from the urogenital tract contribute to the urinary EV repertoire is yet to be established, but it has been shown that several prostate-related proteins and their mRNAs, such as PAP, PSA, prostate-specific membrane antigen (PSMA), prostate stem cell antigen (PSCA), protein–glutamine gamma-glutamyltransferase 4 (TGM4) or transmembrane protease serine 2 (TMPRSS2), are found in urinary EVs [72, 91–93].

Blood-derived EVs have also been extensively investigated in biomarker studies. Blood is a rich source of EVs, but also contains structures that can possibly co-isolate with EVs and mask or disturb EV analyses such as cells, cell-free DNA and lipoproteins. Hence, the isolation and characterisation of blood-derived EVs with high purity is not straightforward. Blood EVs are mainly derived from platelets, red blood cells and leucocytes, as indicated by specific markers of these cell types, CD41, CD235a and CD45, respectively [94]. Blood may be especially relevant for patients with metastatic PCa, considering the distal location of the advanced metastasis of PCa (often bone metastasis) and that many patients with metastatic PCa may have undergone RP. It is not clear how prostate-derived EVs reach the blood circulation. PSA, which is normally secreted from prostate epithelial cells into prostatic fluid, can reach the blood circulation and shows increased serum levels in PCa and other prostatic diseases. This is probably due to morphological and functional changes of prostate and endothelial cells, resulting in increased permeability and leakage of the tumour vasculature, which facilitates the entrance of PSA into blood [95]. EVs are larger in size than PSA, but they may reach the blood system by a similar mechanism. Finally, a recent analysis of the literature of EVs and PCa, including articles with 50 or more patients from 2010 to 2017 (13 articles), showed that almost 30% of the analyses were performed with blood, while in the rest, urine was the selected biofluid [68].

### Preanalytical considerations in EV-biomarker research

Determining inclusion patient criteria for identification of EV-based PCa biomarkers depends on the main purpose of such biomarkers, whether early diagnosis, AS, prognosis or cancer recurrence. Correct sample-size determination is vital if robust conclusions are going to be drawn from EV-biomarker studies. In any case, patient information should be carefully reported, including at a minimum gender, age and clinical-relevant information. In some studies, it may also be important to include additional information such as diet, ethnicity, body mass index, medication, food and fluid intake. In addition, it is fully recognised today that after collection of the biofluid of choice, preanalytical variables should be carefully controlled to avoid degradation before being used for EV-biomarker analysis. Preanalytical variables such as collection method, volume of sample, preservatives, processing and storage temperature can influence the results [83, 96–99], and it is, therefore, essential to report these conditions in detail. To facilitate this, a possibility is to use the Standard PREanalytical Code (SPREC), a seven-element code corresponding to the most critical preanalytical variables of biospecimens [100, 101].

Optimal parameters for the study of EVs in urine and blood (plasma is usually preferred to serum [98]), the two more relevant biofluids for PCa, are being investigated by the respective task forces of the International Society for Extracellular Vesicles (ISEV) [83, 97]. For blood, the fasting status of the donors and the choice of anticoagulant during collection are especially important, and the degree of haemolysis and levels of residual platelets in platelet-free plasma should be measured before using the samples for EV analysis [98, 99]. Concerning the latter, platelets need special attention when studying blood EVs because they can be easily activated under blood collection, handling and storage, and release EVs that may confound the results [99, 102–105]. Two subsequent centrifugations at 2500 × *g* for 15 min have often been used to deplete platelets from plasma samples, but a protocol using a single-step centrifugation has recently been proposed [98, 102, 106]. Moreover, blood samples contain lipoproteins of similar sizes to EVs [35, 107]. When working with blood EVs, separation of lipoproteins is necessary as they are found to be more abundant (100-fold than EVs) in plasma and may confound EV analysis. Combination of methodologies such as ultracentrifugation followed by density gradient or size-exclusion chromatography can improve the purity of EV samples [108].

Urine is a biofluid in close anatomical proximity with the prostate through the prostatic urethra, and it has been the biofluid of choice in several recent studies of EV-based PCa biomarkers (Tables 2 and 3). In these studies, both urine and DRE urine have been used. As mentioned above, DRE urine is a rational choice if high amounts of EV molecules of prostatic origin are needed or if the analyte under investigation has a relatively low abundance [60]. The physiological characteristics of urine and its dynamic character as an excretory biofluid require specific preanalytical steps to assure consistent analysis and experimental results. Timely urine pre-clearing (within hours after collection) by mild centrifugation to remove shed cells is important to prevent cell lysis, which could contaminate the EV fraction with cell debris. If the precleared urine is not to be processed immediately for EV analysis, which is often the case in biobanking and in large clinical studies, it is warranted to store the precleared fraction in aliquots at temperatures below −70 °C [83]. Removal of uromodulin (also known as Tamm–Horsfall protein), a high-abundance protein in urine, has also been the focus of several studies because it forms polymer networks that can trap EVs and skew downstream analysis [109–111]. Urine composition is highly variable (pH, osmolality and concentration) and influenced by certain medications and diet, therefore, an assessment of urine-sample characteristics using dipsticks (e.g., proteins, glucose, ketones, haemoglobin, nitrite, leucocytes and pH) can provide an easy and inexpensive quality-control measure to identify deviating samples. In addition, microbial presence (endogenous, pathological or caused by contamination during sample collection) should also be taken into consideration as it can influence not only EV quantitation, but also the normalisation of experimental data.

**Table 2:** Prostate Cancer Extracellular Vesicles as Diagnostic Biomarkers.

Biomarker	Biofluid	EV isolation	Target detection	Number of patients	Comparison	Performance	Ref.
**mRNAs**							
PCA3 ERG SPDEF	Urine	Urine clinical sample concentration kit (Exosome diagnostics)	RT-qPCR	195 men at initial biopsy	GS ≤ 6 vs. GS ≥ 7	RNAs + SOC AUC 0.8	[157]
				Men undergoing biopsy: Training set: 255 Testing set: 519		Training set: mRNAs + SOC AUC 0.77 Testing set: RNAs + SOC AUC 0.73	[155]
				519 men at initial biopsy		RNAs + SOC AUC 0.71	[159]
PCA3 ERG SPDEF GATA2	Urine	Ultracentrifugation	RT-qPCR	Men undergoing initial biopsy: Training set: 52 Testing set: 165	PCa vs. healthy	RNAs + SOC AUC: Training set: 0.88 Testing set: 0.72	[161]
					GS ≤ 6 vs. GS ≥ 7	RNAs + SOC AUC: Training set: 0.9 Testing set: 0.75	
PCA3 PRAC	Urine	Ultracentrifugation	RT-qPCR	89 men undergoing biopsy	PCa vs. healthy	AUC 0.723	[127]
					GS ≤ 6 vs. GS ≥ 7	AUC 0.736	
PCA3 PCGEM1	Urine	Exosome RNA isolation kit (Norgen)	RT-qPCR	271 men undergoing RP	GS ≤ 6 vs. GS ≥ 7	RNAs + SOC AUC 0.875	[162]
BIRC5 ERG PCA3 TMPRSS2:ERG TMPRSS2	Urine	100 K MWCO filtration concentrator (Millipore)	RT-qPCR	47 PCa 19 healthy men	PCa vs. healthy	BIRC5 AUC 0.674 ERG AUC 0.785 PCA3 AUC 0.681 TMPRSS2:ERG AUC 0.744 TMPRSS2 AUC 0.637	[155]
CDH3	Urine	Ultracentrifugation	Illumina gene expression microarray, RT-qPCR	Discovery cohort: 6 PCa, 4 healthy men Validation cohort: 9 PCa, 7 BPH	PCa vs. BPH	Percentage of samples where CDH3 was detected: BPH 77.78%, PCa 28.57%	[163]
		Norgen exosomal RNA purification kit		Validation cohort: 18 PCa, 7 BPH		CDH3 level significantly decreased in PCa (p 0.01)	
AGR2 splice variants	Urine	Ultracentrifugation	RT-qPCR	24 PCa 15 BPH	PCa vs. BPH	AGR2 SV-H AUC 0.96 AGR2 SV-G ACU 0.94 AGR2 WT AUC 0.91	[164]
**miRNAs**							
miR-21 miR-574 miR-375	Serum	Total exosome isolation kit (Invitrogen)	RT-qPCR	10 healthy men 6 PCa post-RP 8 mPCa	PCa vs. post-RP vs. healthy men	PCa vs. healthy men: miR-21 increased 2-fold miR-574 increased 4-fold miR-375 increased 8-fold Post-RP patients showed intermediate values	[167]
miR-21 miR-200c let-7a	Plasma	SEC	RT-qPCR	50 PCa 22 BPH	PCa vs. BPH	miR-21 AUC 0.67 miR-200c AUC 0.68	[168]
					GS ≤ 6 vs. GS ≥ 8	let-7a AUC 0.68	
miR-574 miR-141 miR-21	Urine	Lectin induced agglutination	RT-qPCR	35 PCa 35 healthy men	PCa vs. healthy	miR-574 AUC 0.85 miR-141 AUC 0.86 miR-21 AUC 0.65	[169]
miR-21 miR-375 let-7c	Urine	Ultracentrifugation	RT-qPCR	60 PCa 10 healthy men	PCa vs. healthy	miR-21 AUC 0.713 miR-375 AUC 0.799 let-7c AUC 0.679	[170]
miR-21 miR-200c	Urine	miRCURY exosome isolation kit (Exiqon)	RT-qPCR	30 non-mPCa 30 mPCa 20 BPH	Non-mPCa vs. mPCa vs. BPH	miR-21 increased in non-mPCa (*p* = 0.001) and mPCa (*p* = 0.018) vs. BPH miR-200c decreased in non-mPCa (*p* = 0.001) and mPCa (*p* = 0.018) vs. BPH	[128]
					mPCa vs. non-mPCa	miR-21 decreased in mPCa (*p* = 0.037)	
miR-375 miR-451a miR-486-3p miR-485-5p	Urine	Exoquick-TC (Systems biosciences)	NGS RT-qPCR	Discovery cohort: 6 PCa 3 healthy men Validation cohort: 47 PCa 29 BPH 25 healthy men	PCa vs. healthy	miR-375 AUC 0.788 miR-451a AUC 0.757 miR-486-3p AUC 0.704 miR-486-5p AUC 0.796	[139]
					PCa vs. BPH	miR-375 + miR-451a AUC 0.726	
					Localised vs. mPCa	miR-375 AUC 0.726	
miR-21 miR-204 miR-375	Urine	Ultracentrifugation	NGS RT-qPCR	Discovery cohort: 9 PCa, 4 healthy men Validation cohort: 48 PCa, 26 healthy men	PCa vs. healthy	isomiRs AUC 0.821	[173]
miR-141	Serum	Exoquick (Systems biosciences)	RT-qPCR	31 non-mPCa 20 mPCa 40 healthy men	PCa vs. healthy	miR-141 significantly increased in PCa (*p* < 0.0001)	[174]
					Non-mPCa vs. mPCa	miR-141 AUC 0.869	
miR-141 miR-125	Plasma	ExoEasy maxi kit (Qiagen)	RT-qPCR	31 PCa 19 healthy men	PCa vs. healthy	miR-125/miR-141 AUC 0.793	[175]
miR-107 miR-574	Plasma	Filtration and concentration	Microarray RT-qPCR	Discovery cohort: 79 PCa, 28 healthy men Validation cohort: 55 PCa, 28 healthy men	PCa vs. healthy	Both miRNAs significantly increased in PCa (*p* < 0.05)	[179]
	Urine		RT-qPCR	135 men after DRE		miR-107 AUC 0.74 miR-574 AUC 0.66	
miR-145	Urine	Hydrostatic filtration dialysis, ultracentrifugation	RT-qPCR	60 PCa 37 BPH 24 healthy men	PCa vs. BPH	miR-145 + PSA AUC 0.86	[176]
miR-2909	Urine	miRCURY Exosome Isolation Kit (Exiqon)	RT-qPCR	90 PCa 10 BPH 60 bladder cancer 50 healthy men	GS ≤ 6 vs. GS 7 vs. GS ≥ 8	miR-2909 significantly increased in GS 7 compared to GS 6 and in GS 8 compared to GS 7 (*p* < 0.001)	[177]
miR-196a miR-501	Urine	Ultracentrifugation	NGS RT-qPCR	Discovery cohort: 20 PCa, 9 healthy men Validation cohort: 28 PCa, 19 healthy men	PCa vs. healthy	miR-196a AUC 0.73 miR-501 AUC 0.69	[180]
miR-30b miR-126	Urine	Ultracentrifugation	Microarray RT-qPCR	Discovery cohort: 10 PCa, 4 healthy men Validation cohort: 28 PCa, 25 healthy men	PCa vs. healthy	miR-30b AUC 0.663 miR-126 AUC 0.664	[181]
miR-23b miR-27a miR-27b miR-1 miR-10a miR-423	Urine	Acoustic trapping	NGS	147 PCa 60 healthy men	GS ≤ 8 vs. GS ≥ 9	miR-23b *p* = 0.0033 miR-27a *p* = 0.0027 miR-27b *p* = 0.0136 miR-1 *p* = 0.0037 miR-10a *p* = 0.0239 miR-423 *p* = 0.0271	[144]
miR-1246	Serum	Total exosome isolation reagent (Life technologies)	Nanostring nCounter microarray, RT-qPCR	Discovery cohort: 6 PCa, 3BPH, 3 healthy Validation cohort: 44 PCa, 4 BPH, 8 healthy	PCa vs. BPH	miR-1246 significantly increased in PCa (*p* = 0.0041)	[178]
					PCa vs. healthy	miR-1246 AUC 0.926	
miR-142-3p miR-142-5p miR-223	Semen	Ultracentrifugation	RT-qPCR	24 PCa 7 BPH 8 healthy men	PCa vs. BPH + healthy men	miR-142-3p AUC 0.739 miR-142-5p AUC 0.733 miR-233 AUC 0.722	[183]
					PCa vs. BPH	miRNAs + PSA AUC 0.821	
miR-142 miR-196b miR-30c miR-34a miR-92a	Semen	Ultracentrifugation	RT-qPCR	9 PCa 5 BPH 12 healthy men	PCa vs. healthy men	miR-142, miR-196b, miR-30c and miR-34a significantly different (*p* < 0.05)	[184]
		miRCURY Exosome Cell/UrineCSF Kit (Qiagen)				No significant differences found	
		ExoGAG (NasasBiotech)				miR-142 and miR-92a significantly different (*p* < 0.05)	
**Proteins**							
PSA	Plasma	Ultracentrifugation	ELISA	15 PCa 15 BPH 15 healthy men	PCa vs. BPH *vs*. healthy men	PSA expression was 4.5–5 times higher in PCA than in healthy men and BPH	[185]
				80 PCa, 80 BPH, 80 healthy men	PCa vs. BPH	PSA AUC 1	[186]
					PCa vs. healthy	PSA AUC 0.98	
TGM4 ADSV PSA PPAP CD63 SPHM GLPK5	Urine	Ultracentrifugation	SRM-proteomics	22 PCa low risk (GS 3 + 4 or lower) 31 PCa high risk (GS 4 + 3 or higher) 54 healthy men	PCa vs. healthy	TGM4 + ADSV AUC 0.65	[187]
					PCa low vs. PCa high risk	PPAP + PSA + CD63 + SPHM + GLPK5 AUC 0.7	
CD9, CD63, PSA	Urine	Ultracentrifugation	TR-FIA	67 PCa 76 healthy men	PCa vs. healthy	CD63/PSA AUC 0.68 CD9/PSA AUC 0.61	[81]
CD9	Plasma	Ultracentrifugation	TR-FIA	6 PCa 10 BPH	PCa vs. BPH	CD9 significantly increased in PCa (*p* = 0.0291)	[188]
Surface proteins	Plasma	CD13 capture	Proximity ligation assay, qPCR	Two cohorts: 20 PCa, 20 healthy men 13 PCa, 13 healthy men	PCa vs. healthy	PCa signal significantly higher in both cohorts (*p* < 0.001)	[190]
				20 GS ≤ 6 19 GS 7 20 GS 8–9	GS ≤ 6 vs. GS 7 vs. GS 8–9	GS 7 and GS 8–9 significantly higher signal than GS 6 (*p* < 0.001). No significant difference between GS 7 and GS 8–9.	
Survivin	Plasma	Ultracentrifugation	Western blot, ELISA	28 PCa 6 healthy men	PCa vs. healthy	Survivin significantly increased in PCa (*p* < 0.05)	[191]
	Serum	Exoquick (Systems Biosciences)		19 PCa, 20 BPH, 10 healthy men	PCa vs. BPH vs. healthy men	Survivin significantly increased in PCa compared to both BPH and healthy (*p* < 0.001)	
	Serum	Exoquick (Systems Biosciences)	ELISA	17 PCa (European American) 21 PCa (African American) 10 healthy men	PCa (European American) *vs*. PCa (African American) vs. healthy men	Survivin significantly higher in both PCa populations compared to healthy men (*p* < 0.001). Survivin significantly increased in African American patients (*p* < 0.001)	[192]
	Plasma			10 PCa (European American) 12 PCa (African American)	PCa (European American) vs. PCa (African American)	Survivin significantly increased in African American patients (*p* < 0.001)	
TMEM256 LAMTOR1	Urine	Ultracentrifugation	MS	16 PCa 15 healthy men	PCa vs. healthy	TMEM256 + LAMTOR1 AUC 0.94	[93]
FABP5	Urine	Ultracentrifugation	LC-MS/MS, SRM	Discovery cohort: 6 PCa GS 6, 9 PCa GS 8–9, 6 healthy men Validation cohort: 5 PCa GS 6, 13 PCA GS ≥ 7, 11 healthy men	PCa vs. healthy	FABP5 AUC 0.757	[193]
					GS ≤ 6 vs. GS ≥ 7	FABP5 AUC 0.856	
PTEN	Plasma	Ultracentrifugation	Western blot	30 PCa 8 healthy men	PCa vs. healthy	PTEN detected only in EVs from PCa patients	[194]
Flot2 Park7	Urine	Ultracentrifugation	ELISA	26 PCa 16 healthy men	PCa vs. healthy	Flot2 AUC 0.65 Park7 AUC 0.71	[195]
EphrinA2	Serum	Ultracentrifugation	ELISA	50 PCa (19 GS 6–7, 31 GS 8–9; 18 T1-T2, 32 T3-T4) 21 BPH 20 healthy men	PCa vs. BPH vs. healthy	EphrinA2 AUC 0.766	[196]
					GS 6–7 vs. GS 8–9 T1-T2 vs. T3-T4	EprhinA2 level increased in GS 8–9 compared to GS 6–7 (p = 0.02) and in T3-T4 compared to T1-T2 (*p* = 0.03)	
Del-1	Serum	CD63 capture	ELISA	276 PCa 182 benign	PCa vs. BPH	Del-1 AUC 0.68	[197]
ITGA3 ITGB1	Urine	Ultracentrifugation	Western blot	5 non-mPCa 3 mPCa 5 BPH	mPCa vs. non-mPCa vs. BPH	Both proteins significantly increased in mPCa: ITGA3 (*p* < 0.005) ITGB1 (*p* < 0.01)	[198]
GGT1	Serum	Ultracentrifugation	Protein activity with Proteo-GREEN-gGlu	31 PCa 8 BPH	PCa vs. BPH	GGT1 activity increased in PCa EVs (*p* < 0.05), but not when measured directly in serum	[199]
STEAP1	Plasma		Nanoscale flow cytometry	121 PCa 55 healthy men	PCa vs. healthy	STEAP1 AUC 0.95	[200]
**Other EV molecules or quantification methods**							
LacCer(d18:1/16:0), PS 18:1/18:1, PS 18:0/18:2	Urine	Ultracentrifugation	MS	15 PCa 13 healthy men	PCa vs. healthy	Lipid combination AUC 0.989	[201]
Metabolites profile	Urine	Ultracentrifugation	UHPLC-MS	31 PCa 14 BPH	PCa vs. BPH	76 metabolites differentially expressed between PCa and BPH (*p* < 0.05)	[202]
lncRNA-p21	Urine	Urine exosome RNA isolation kit (Norgen)	RT-qPCR	30 PCa 49 BPH	PCa vs. BPH	lncRNA-p21 AUC 0.663	[204]
SAP30L-AS1, SChLAP1	Plasma	Total exosome isolation reagent (Invitrogen) followed by immunoaffinity	RT-qPCR	34 PCa 46 BPH 30 healthy men	PCA vs. BPH and healthy men	SAP30L-AS1 AUC 0.65 SChLAP1 AUC 0.87 Both RNAs AUC 0.92	[205]
sncRNA profile (miR Sentinel Test)	Urine	Urine exosome RNA isolation kit (Norgen)	Affimetrix geneChip miRNA 4.0 array	Discovery cohort: 146 PCa (90 grade 1, 34 grade 2, 9 grade 3, 7 grade 4, 6 grade 5) 89 healthy men Validation cohort: 868 PCa (437 grade 1, 162 grade 2, 131 grade 3, 66 grade 4, 72 grade 5) 568 healthy men	PCa vs. healthy	Sensitivity 94% and specificity 92%	[206]
					ISUP grade 1 vs. ISUP grade 2–5	Sensitivity 93% and specificity 90%	
					ISUP grade 1–2 vs. ISUP grade 3–5	Sensitivity 94% and specificity 96%	
Vesicle amount	Serum	Ultracentrifugation	Microfluidic raman biochip	10 PCa 8 healthy men	PCa vs. healthy	Number of vesicles significantly increased in PCa (*p* < 0.0001)	[208]

Only studies with over 10 individuals were included.

*AUC* area under the curve, *BPH* Benign prostate hyperplasia, *DRE* digital rectal exam, *EVs* extracellular vesicles, *GS* Gleason score, *LC* liquid chromatography, *mPCa* metastatic prostate cancer, *MS* mass-spectrometry, *MWCO* molecular weight cut off, *PCa* prostate cancer, *RP* radical prostatectomy, *SEC* size-exclusion chromatography, *SOC* standard of care, *SRM* selective reaction monitoring, *TR-FIA* time-resolved fluorescence immunoassay, *UHPLC* ultra high performance liquid chromatography, *vs.* versus.

**Table 3:** Prostate cancer extracellular vesicles as prognostic and monitoring biomarkers.

Biomarker	Biofluid	EV isolation	Target detection	Number of patients	Comparison	Performance	Ref.
**mRNAs**							
AR-V7	Plasma	ExoRNeasy kit (Qiagen)	ddPCR	36 mCRPC before second-line hormonal treatment	AR-V7^+^ vs. AR-V7^-^	PFT 3 vs. 20 months, OS not reached vs. 8 months	[221]
				9 CRPC, 7 HSPC 5 healthy men	PCa vs. healthy	Similar level of AR-V7 expression in EVs	[222]
		Exoquick (System Biosciences)		35 CRPC	AR-V7^+^ vs. AR-V7^-^	PFT 16 vs. 28 months	[219]
AR-V7/AR-FL ratio	Urine	Exo-Hexa	ddPCR	22 HSPC, 14 CRPC 11 healthy men	CRPC vs. HSPC	AUC 0.87	[223]
	Plasma	ExoRNeasy kit (Qiagen)		73 CRPC	AR-V7^+^ vs. AR-V7^-^	PFS 4 vs. 20 months OS not reached vs. 9 months	[218]
CD44v8–10	Serum	ExoRNeasy kit (Qiagen)	ddPCR	50 docetaxel naive 10 docetaxel resistant 15 healthy men	Docetaxel resistant vs. docetaxel naive	46 vs. 12 copies/ml (*p* = 0.032)	[226]
					Docetaxel resistant vs. healthy men	46 vs. 17 copies/ml (*p* = 0.032)	
BRN4 BRN2	Serum	Total exosome isolation reagent (Life Technologies)	RT-qPCR	42 mCRPC 6 mCRPC with NED	mCRPC-NE vs. mCRPC	Higher levels of BRN4 and BRN2 in mCRPC-NE: EV-BRN4 AUC 1 EV- BRN2 AUC 0.944	[228]
				42 mCRPC 6 mCRPC with NED 23 CRPC	mCRPC with enz. vs. mCRPC wo enz.	EV-BRN4 FC ≈ 7 (*p* < 0.0001) EV-BRN2 FC ≈ 4 (*p* < 0.0001)	
CK-8	Plasma	ExoRNeasy kit (Qiagen)	RT-qPCR	62 mCRPC 10 healthy men	Positive vs. negative	OS 16.9 vs. 31.8 months (*p* = 0.001)	[210]
LASSO criteria (36 different mRNAs)	Urine	Microfiltration	Nanostring expression	Discovery cohort: 535 PCa Diagnostic cohort: 177 PCa Prognostic cohort: 87 PCa	D’Amico classification (normal vs. low vs. medium vs. high risk)	Model predicted the presence of clinically significant intermediate‐ or high‐risk disease. AUC 0.77	[214]
						Able to detect BCR with HR 2.86 (*p* < 0.001)	
**miRNAs**							
miR-375 miR-1290	Plasma	Exoquick (System Biosciences)	NGS RT-qPCR	Discovery cohort: 23 mCRPC Validation cohort: 100 mCRPC	High vs. low miR-375 and miR-1290 levels	OS 7.23 vs. 19.3 months miRNAs + PSA + ADT failure time predict OS with AUC 0.73	[209]
miR-141 miR-375	Serum	ExoMiR extraction kit (Bioo Scientific)	RT-qPCR	47 recurrent PCa, 72 non-recurrent PCa	Recurrent PCa vs. non-recurrent PCa	Increased levels in metastasis (*p* < 0.0001)	[179]
miR-151a miR-204 miR-222 miR-23b miR-331 PSA	Urine	miRCury exosome isolation kit (Qiagen)	RT-qPCR	Discovery cohort: 215 RP Validation cohorts: Cohort 2: 199 RP Cohort 3: 205 RP	Pre- vs. post- RT	Predictor of BCR Discovery: HR 3.12, (*p* < 0.001) Cohort 2 HR 2.24 (*p* = 0.002) Cohort 3: HR 2.15 (*p* = 0.004)	[215]
**Proteins and other molecules or EV quantification methods**							
ACTN4	Serum	Ultracentrifugation	Proteomic analysis	36 PCa (8 untreated, 8 ADT, 20 CRPC different therapies)	CRPC vs. ADT	FC 1.4 (*p* < 0.01)	[227]
GSTP1 and RASSF1A methylation	Plasma	ExoRNeasy kit (Qiagen)	RT-qPCR	62 mCRPC 10 healthy men	Positive vs. negative	GSTP1 OS 8.6 vs. 21.4 months (*p* = 0.015) RASSF1A OS 8.0 vs. 22.6 months (*p* = 0.007)	[210]
Vesicle amount	Serum	Total exosome isolation kit (Invitrogen)	RT-qPCR	11 PCa GS ≥ 7	Post- vs. pre-RT	FC 1.3 (*p* < 0.52)	[225]
	Plasma	Antibody-captured	Nanoscale FACS	265 PCa, 67 mCRPC, 156 BPH, 22 healthy men	mCRPC vs. PCa	Higher levels of PSMA^+^ EVs in mCRPC	[211]
					GS ≤ 7 vs GS ≥ 8	Higher levels of PSMA^+^ EVs in GS ≥ 8	
				25 mCRPC	Pre-RP vs. post-RP	Higher levels PSMA^+^ EVs in pre-RP	
	Blood	Antibody-captured	ACCEPT software image analysis	190 CRPC	Low vs. high amount of EVs	OS 31.6 vs. 14.7 months HR 2.2 (*p* = 0.001)	[213]
	Blood	Antibody-captured	ACCEPT Software image analysis	Discovery: 84 mCRPC Validation: 45 mCRPC 93 Healthy men	Low vs. high amount of EVs	OS 23 vs. 8.1 months (*p* < 0.001) HR 3.8 (*p* < 0.001)	[212]

Only studies with more than 10 individuals were included in the table.

*ADT* androgen-deprivation therapy, *AR* androgen receptor, *AUC* area under the curve, *BCR* biochemical recurrence, *BPH* Benign prostate hyperplasia, *CI* confidence interval, *CRPC* Castration-resistant prostate cancer, *CTCs* circulating tumour cells, *ddPCR* digital-droplet PCR, *enz.* Enzatulamide, *EV* extracellular vesicles, *FL* full-length, *GS* Gleason score, *HR* Cox hazard ratio, *HSPC* hormone-sensitive prostate cancer, *mCRPC* metastatic castration-resistant prostate cancer, *NED* neuroendocrine differentiation, *OS* overall survival, *PCa* prostate cancer, *PFS* progression-free survival, *PSA* prostate-specific antigen, *RP* radical prostatectomy, *RT* radiation therapy, *v7* Variant 7, *vs.* versus, *wo* without.

### Conventional and novel methodological approaches for the analysis of EVs in liquid biopsies

In early days, the most common method to isolate EVs was differential centrifugation, and the smaller EV population was enriched by ultracentrifugation (often at 100,000 × *g*) for 1–2 h [112]. Today, a plethora of methods based on different physical and molecular EV characteristics are available, including filtration, precipitation, hydrostatic dialysis, ultrafiltration, size-exclusion chromatography, immunocapture and acoustic trapping [113–119]. Moreover, a combination of different isolation methods can also be an option in some cases. Considering the diverse methodology available for EV separation, it is important to be aware of the advantages and disadvantages of the different methods, which have been presented in numerous publications [59, 114, 115, 117–120]. For example, when working with biofluids, it can be an advantage to use immunocapture with a cancer-related or a tissue-specific molecule because biofluids contain several EV populations that can mask the signal of the EV population of interest.

As shown in Tables 2 and 3, several EV-isolation methods have been used to separate EVs from biofluids to identify PCa biomarkers. A challenge in EV isolation is that different isolation methods may lead to different results, probably because the methods separate to different degrees the different types of EVs and other molecular structures present in the sample [121–124]. Moreover, it is not always practical to use some of these methods in a clinical setting for different reasons, such as low throughput, requirement of a large amount of sample or expensive and difficult-to-use instrumentation. Indeed, several easy-to-use isolation kits have been commercialised. Although these methods could be very useful in a clinical setting, a main drawback is that the isolation principle, the kit components and how they affect other structures in the biofluid are often not clearly specified [123]. Careful consideration of the pros and cons of each method, the availability of starting material and the downstream analysis, is needed to determine the most suitable methodology for the isolation of EVs from biofluids. In fact, it should be considered if it is necessary to separate EVs from the biofluid because isolation protocols often lead to EV loss and can be biased towards an EV population. Direct and rapid analyses of EVs in biofluids would be an advantage for clinical implementation [83].

The molecular content of EVs shows a large diversity, but the search for novel PCa EV biomarkers has focused mainly on the analysis of proteins, mRNAs, lncRNAs and miRNAs in EVs isolated from urine or blood. Standard analytical methods to analyse the molecule of interest, such as immune-based methods for protein analysis and PCR for nucleic acid analysis, have often been used (Tables 2 and 3). In addition, several omics methods allowing simultaneous analysis of many molecules, i.e., mass spectrometry (MS) and next-generation sequencing, have also been very useful for identifying novel EV biomarkers for PCa [83]. Moreover, changes in EV numbers are also being investigated as a PCa biomarker. For EV-biomarker analysis, the normalisation method should be carefully chosen to obtain solid results. Several normalisation methods have been used when analysing EVs in liquid biopsies for prostate cancer, such as the levels of urinary PSA, the number of vesicles or the total vesicle-protein amount [81, 83]. There is not a universal normalisation method for the results of EV experiments, and the ideal normalisation method depends on the biofluid, sample handling and target molecule. Working with urine requires additional care because the concentration of EVs in this biofluid is affected by the overall urine concentration, which shows great inter- and intra-patient variability. A recent study has shown that the levels of creatinine, which is commonly used to normalise soluble urinary biomarkers, are highly correlated with the number of EVs [125]. The same study also reported that the addition of uromodulin affects the particle counts. It is also important to consider that the preparation and analysis of EVs is a potential source of variability. In order to account for this, trackable recombinant EVs have recently been developed [126, 127]. Spiking this or other reference materials in biofluids can be used to normalise for technical errors during sample preparation and analysis between samples. Finally, the normalisation of molecular data is also a challenge. For example, several strategies have been developed for the normalisation of RNA results [126]. The results of some studies have been normalised to the level of one or several reference transcripts [127–129]. An interesting alternative is the use of the geometric mean of all the studied RNA species [130]. Finally, adding a synthetic spike-in RNA during different stages of the RNA analysis can be a helpful tool to avoid bias caused by library preparations or PCR efficiencies [131].

Since the different areas of EV research have different demands in terms of EV isolation, some recent articles have focused on the isolation and analysis of EVs from biofluids using novel technologies such as microfluidic, nanotechnology and label-free approaches [132–134]. For example, microfluidic EV-isolation technologies have gradually emerged in the last few years, having the potential to overcome many of the drawbacks associated with conventional isolation techniques [135]. These techniques offer several benefits such as low sample volumes, low costs, high precision and automation. The advances in nanofabrication and the possibility to integrate nanomaterials to enhance the performance of the devices can provide unprecedented opportunities in the biosensing field [134, 136–138]. Further, the integration of isolation of EVs with their detection and analysis on the same platform can boost the next generation of point-of-care devices. Microfluidic EV-isolation techniques are generally based on EV-surface markers (immunoaffinity capture) or physical characteristics of EVs such as size, charge or density [107, 117, 139, 140]. Immunoaffinity relies on the use of antibodies (or beads coated with antibodies) against EV-surface proteins. The most commonly used antibodies target tetraspanin proteins such as CD63 or CD9, which are generally enriched in EV membranes. Besides, EVs from different cell origin can be selectively recognised by using antibodies against molecules overexpressed in cancer cells [141]. On the other side, EVs can be isolated, depending on their physical properties. Nanoscale deterministic lateral-displacement pillar arrays are an efficient technology to sort and separate EVs, because EVs follow different trajectories in a pillar array depending on their size [142]. When integrating these arrays on a chip, a superior yield of EVs was isolated from serum and urine compared with conventional isolation techniques such as ultracentrifugation or density-gradient ultracentrifugation [143]. Ultrasonic waves can also be used to isolate and enrich EVs, enabling downstream small RNA sequencing from PCa clinical samples [144]. In addition, electrostatic interactions were used as separation principle in a nanowire-anchored microfluidic device that also allowed in situ extraction of RNA [140]. When applied to urine samples, the device showed higher efficiency of miRNA extraction and a much larger variety of miRNAs than ultracentrifugation. However, the positively charged surface nanowires have low selectivity in terms of EV analysis because they collect indiscriminately negatively charged structures in urine, including EVs and free negatively charged molecules such as miRNAs [140]. Another technology that has been described to separate EVs in a size-dependent and label-free manner is viscoelasticity-based microfluidics, showing a high level of recovery (>80%) and purity (>90%) of EVs [145]. Similarly, sheathless oscillatory viscoelastic microfluidics has been used to separate EVs, although further research is needed to bring these technologies into the clinics [146].

In addition to EV isolation, the possibility to integrate EV detection and analysis within the same platform is gaining considerable attention. Combining microfluidics with techniques, such as fluorescence, surface plasmon resonance, colorimetric or electrochemical detection, has opened the path towards clinical translation [147]. Pioneering examples of these platforms include the ExoChip device that can isolate EVs directly from blood using a microfluidic device functionalized with anti-CD63 antibodies and quantify them using a fluorescent dye and a plate reader [148]. Going a step further, the ExoSearch chip allows on-chip isolation and multiplexed detection of tumoural EV in 40 min [149]. The integration of these platforms with detection systems or smartphones as imaging read-out systems is emerging as an ideal approach for point-of-care diagnosis due to the excellent portability and cost-effectiveness of these devices [150–153]. Although much effort has been done for the development of portable and automatised devices for the isolation, detection and analysis of EVs, many of the reports are still at a proof-of-concept level [134].

## EV-based biomarkers for prostate cancer

A main aim of the studies of EV-based biomarkers for PCa is to improve detection of clinically significant PCa and aid clinical decision-making for patients within each risk group. Biomarkers can be divided into different categories based on their particular application [154]. In this review, we have classified the identified EV biomarkers into two main groups. In the group of diagnostic biomarkers, we have included the biomarkers used for the detection of PCa and/or the stratification of patients according to GS or ISUP grade (Table 2). The biomarkers that predict survival rates, cancer progression, probability of metastasis and development of treatment resistance or cancer recurrence have been included in the prognostic and monitoring group (Table 3). Only studies containing more than 10 individuals are included in the tables.

### Prostate-cancer extracellular vesicles as diagnostic biomarkers

Studies of diagnostic biomarkers have compared PCa patients with healthy individuals, but also to patients afflicted with benign prostate hyperplasia (BPH), which is also usually related to an increased serum PSA level. Additionally, several publications have addressed the necessary distinction between low-risk PCa, which may not require aggressive treatment, and intermediate- and high-risk prostate tumours that require treatment. Usually GS or the equivalent ISUP grade, together with PSA and clinical stage, is used to classify the PCa risk [3].

In 2009, Nilsson et al. showed that the RNAs PCA3 and TMPRSS2:ERG were found in urinary EVs [71]. Interestingly, the presence or absence of TMPRSS2:ERG in urinary EVs mimics the results from prostate biopsies [155]. While one study claimed that the expression of PCA3 alone in urinary EVs is not a good predictor of PCa [156], others found that PCA3, ERG, BIRC5, TMPRRS2 and TMPRRS2:ERG can differentiate between healthy and PCa patients [155]. The analysis of a cohort of 195 men showed that the expression of PCA3 and ERG genes (including the fusion gene TMPRSS2:ERG) normalised to the level of SPDEF (SAM-pointed domain-containing Ets transcription factor) can be used to differentiate between GS ≤ 6 and GS ≥ 7 tumours [157]. This result was later confirmed in independent cohorts of 519 and 503 patients [158, 159]. These results are the basis of the EV-based ExoDx PCa test, which helps to decide about biopsy for men over the age of 50 and PSA 2–10 ng/ml [160]. In the first study from 2009, sequential centrifugation was used to isolate EVs from both DRE- and non-DRE urine [71]. Later studies have used non-DRE urine and ultrafiltration centrifugation to concentrate the vesicles and detect PCA3 and ERG [158, 159]. Additionally, a recent independent study including 217 men proposed that the addition of GATA2 to this model could improve the detection of high-risk PCa [161]. Further studies with urinary EVs have reported that the ratio between PCA3 and PCa-susceptibility candidate (PRAC) can differentiate both between healthy men and PCa patients and between GS ≤ 6 and GS ≥ 7 in a cohort of 89 individuals [127] and that PCA3, together with PCGEM1, can be used to distinguish between favourable and unfavourable intermediate tumours (GS 3 + 4 vs GS 4 + 3 or higher) in a racially diverse cohort of 271 patients [162]. Analysis of a microarray panel identified a decrease in CDH3-expression level in PCa patients compared with BPH in independent cohorts using different EV-isolation methods [163]. The different AGR2 splice variants can also distinguish between BPH and PCa [164].

Several miRNAs previously identified as PCa biomarkers have been detected in EVs. miR-21 is one of the most commonly identified [165, 166]. Li et al. compared the expression of miR-21, miR-574 and miR-375 in serum EVs of treated and untreated PCa patients as well as healthy men, and showed that the miRNAs levels were higher in untreated patients than in healthy donors, while patients after RP showed an intermediate level [167]. Later studies have confirmed the increase in miR-21 levels in PCa patients compared with healthy individuals or BPH patients in plasma [168] and urine [128, 169, 170]. Other prominent miRNAs previously detected in liquid biopsies for PCa and later identified in EVs are miR-375 and miR-141 [171, 172]. miR-375 was also found differentially expressed between PCa patients and healthy donors in urinary EVs in a cohort of 70 men [170], and was also selected in an independent discovery cohort [129]. Interestingly, one study could not find differences in miR-21 or miR-375 levels in urinary EVs, but detected a significant change in the expression of their corresponding isomiRs [173]. miR-141 has also been found to be deregulated in EVs in both urine [169, 174] and plasma [175]. A few other miRNAs previously related to PCa have also been validated in urinary EVs, such as miR-145 [176], miR-2909 [177] and miR-200c [128].

Several studies have been designed to identify novel EV miRNAs for PCa diagnosis. miR-1246 was found significantly altered in the serum of PCa patients [178]. In addition, miR-574 and miR-107 have been identified in plasma samples as PCa biomarkers [179]. These miRNAs showed a similar behaviour in urinary EVs [169, 179]. Other miRNAs such as miR-196a and miR-501 [180], miR-451a and miR-486 [129] and miR-30b and miR-126 [181] were found to be altered in urinary EVs of PCa patients compared with healthy men. Recently, Ku et al. developed a new technique for urinary EV isolation, acoustic trapping, and detected several miRNAs deregulated in patients with high-risk PCa (grade 3 or lower versus grades 4 and 5) [144]. One of them was miR-23b, which had previously been found to be deregulated in plasma EVs of PCa patients compared with healthy donors [182]. In terms of other biofluids, Barcelo et al. showed that miRNAs found in EVs isolated from semen can also be used as biomarkers in a discovery cohort of 12 patients and in a validation cohort of 39 individuals. They reported that a model including PSA and 3 miRNAs (miR-142-3p, miR-142-5p and miR-223) could differentiate between PCa and BPH patients, while the combination of PSA, miR-342 and miR-374 was able to distinguish GS 6 from GS 7 [183]. The first model was later confirmed using 3 different EV-isolation protocols in an independent cohort of 26 donors [184].

Logozzi et al. studied the potential of PSA associated with plasma vesicles as a biomarker. In a cohort of 45 individuals, the EV–PSA level was higher in PCa patients than in BPH patients or healthy individuals [185]. A follow-up study, including 240 individuals, showed that EV-derived PSA outperforms the conventional PSA test [186]. An MS analysis of urinary EVs also included PSA in a panel of 5 proteins (CD63–GLPK5–SPHM–PSA–PAPP) able to distinguish between low- and high-grade patients [187]. Moreover, the tetraspanins CD63 and CD9 were analysed in DRE urine (100 µl of cell-free urine) using a time-resolved fluorescence immunoassay developed by Duijvesz et al. for capture/detection of PCa-derived EVs. Using this sensitive assay, the expression level of CD63 and CD9, normalised to urinary PSA, was higher in PCa patients than in healthy men [81]. Interestingly, it was also found that the levels of CD9 and CD63 were very low in urine from women, men after prostatectomy and non-DRE urine. Using the same assay, Soekmadji et al. reported that the CD9 level was higher in plasma of PCa patients than in benign patients [188]. Moreover, EV immunocapture with CD13/aminopeptidase N, a protein found in semen EVs [189], was used to develop a proximity-ligand assay using four antibodies attached to DNA strands as analytical method [190]. It was shown then that the signals measured directly in blood samples from PCa patients were higher compared with healthy men. This assay also distinguished patients with GS ≤ 6 from patients with higher GS.

Another protein that has been investigated as PCa biomarker is survivin, a member of the inhibitor of apoptosis family of proteins. The levels of this protein in plasma EVs measured by ELISA were reported to be higher in PCa patients than in BPH patients or healthy individuals [191], and this result was later confirmed in an independent cohort [192]. MS allows the identification of over 1000 proteins simultaneously and has been used for the discovery of novel EV-based PCa biomarkers. For example, MS analysis of urinary EVs from healthy men and PCa patients found several deregulated proteins, including TMEM256 and LAMTOR1 [93]. Another study showed that FABP5 distinguished between healthy individuals and patients with low-risk (GS 6) and intermediate–high-risk PCa tumours [193]. The EV levels of PTEN [194], flotillin 2 and PARK7 [195], ephrinA2 [196], Del-1 [197], the integrins ITGA3 and ITGB1 [198] and GGT1 activity [199] have also been reported to differentiate between PCa patients and healthy individuals and/or BPH patients. In addition, the prostate-enriched protein STEAP1 was found to be increased in plasma samples of PCa patients compared with healthy males [200].

While mRNAs, miRNAs and proteins are the most common molecules studied as PCa biomarkers, some reports highlight the potential of using other types of EV cargos. Skotland et al. found several lipid species in urinary EVs that were differentially expressed in PCa patients and healthy men [201]. Moreover, Clos-Garcia et al. identified 76 lipids and metabolites differentially expressed between PCa and BPH in urinary EVs [202]. Interestingly, urinary EVs seem to reflect several metabolic alterations reported in PCa, including phosphathidylcholines, acyl carnitines and citrate. Puhka et al. have also shown the potential of metabolomics analysis of urinary EVs in PCa [203]. For EV-derived nucleic acid cargo, three long non-coding RNAs have been proposed to differentiate between prostate tumours and BPH: lncRNA-p21 in urine [204] and SAP30L-AS1 and SChLAP1 in plasma [205]. The different miR Scientific’s Sentinel tests use a profile of urinary EV small non-coding RNAs to differentiate between healthy and PCa patients or stratify according to the ISUP grade [206]. Other projects have explored the possibility of using light-scattering techniques for EV analysis. Krafft et al. showed that the Raman spectra of EVs from PCa patients and healthy individuals were different [207], and in another study, the amount of vesicles estimated by spectroscopy was higher in PCa patients than in healthy men [208].

### Prostate-cancer extracellular vesicles as prognostic and monitoring biomarkers

Several studies have reported alterations in the expression levels of EV miRNAs isolated from CRPC patients and their prognostic power. For instance, an increase in miR-1290 and miR-375 levels has been associated with poor overall survival (OS) (7.23 months vs. 19.3 months) [209]. In serum EVs, the expression of miR-375 and miR-141 was able to distinguish recurrent from non-recurrent PCa [179].

Another study performing a direct comparison of DNA-methylation markers and gene expression between paired CTCs and plasma-derived EVs of mCRPC patients showed that CK-8 expression, together with RASSF1A and GSTP1 methylation, correlated with lower OS (16.9 months vs. 31.8 months, 8.0 months vs. 22.6 months and 8.6 months vs. 21.4 months, respectively) [210]. Moreover, when comparing PSMA-positive plasma EV levels in mCRPC patients, BPH patients and healthy men, PSMA-positive EVs were predominant in mCRPC [211]. This result correlates with recent findings by Nanou et al. where a higher amount of tumour-derived EVs were found in the plasma of CRPC patients compared with healthy men, and that an increase in EV numbers was associated with lower OS [212, 213]. Another approach used the RNA expression profiles from urinary EVs to predict cancer progression within 5 years in a cohort of AS patients [214]. RNA profiling also showed that the expression of five miRNAs in EV-enriched urine (miR-151a-5p, miR-204-5p, miR-222-3p, miR-23b-3p and miR-331-3p) and serum PSA levels predicted the time of recurrence after RP in 3 independent cohorts [215].

Several studies have identified biomarkers that could serve as drug-resistance predictors for PCa treatment [216, 217]. Androgen receptor (AR) variants, in particular, the AR-Variant 7 (AR-V7), are of special interest due to their crucial role in CRPC development [218–220]. In 2016, Del Re et al. reported that 36% patients of a CRPC cohort were positive for AR-V7 mRNA in plasma-derived EVs. AR-V7 EV expression correlated with lower mean progression-free survival (20 vs. 3 months) and OS (not reached vs. 8 months) [221]. In contrast, other studies reported that only a minor fraction of plasma-derived EVs from CRPC patients contained AR-V7 and suggested that CTCs might be a better predictor for AR-therapy failure [23, 222]. Higher levels of AR-V7 transcripts have also been shown in urinary EVs derived from CRPC patients compared with hormone-sensitive PCa patients [223].

Among other potential biomarkers, studies analysing EVs in serum of CRPC patients have shown that the tandems miR-654-3p and 379-5p and miR let-7a-5p and miR-21-5p might predict the efficiency of RT [224, 225]. In addition, CD44v8–10 mRNA copy numbers could predict resistance to docetaxel [226]. While comparing serum EV-protein content released by CPRC patients versus localised PCa patients receiving ADT treatment, proteomic analysis revealed that actinin-4 was highly expressed in the CPRC cohort [227]. An interesting study conducted by Bhagirath et al. has shown that enzalutamide treatment increases the release of BRN4 and BRN2 mRNA via serum EVs and that it may modulate the progression from CRPC to neurocrine PCa [228].

Finally, it is plausible that some of the previously identified PCa biomarkers in biofluids are indeed part of EVs, for example, caveolin-1, a membrane protein that plays a role in PCa cell survival [229, 230]. The levels of caveolin in serum have been reported to be higher in PCa patients than in healthy men and men with BPH [231]. In addition, the preoperative level of serum caveolin-1 can predict decreased time to cancer recurrence [232].

## Challenges and possible solutions for the use of EVs in liquid biopsies for prostate cancer

As presented in this review, EVs have actively been investigated in the last decade as potential biomarkers for PCa in liquid biopsies. However, the analysis of EVs in biofluids is not trivial, and several challenges have been found in the translation of EV-based biomarkers into the clinic [233–235]. Table 4 shows the main challenges and possible solutions for the analysis of EVs in liquid biopsies for PCa. For example, a main challenge has been the initial lack of methodological consensus and reporting in the EV field, now addressed by several initiatives such as MISEV and EV-track [31, 236]. Another hurdle that still needs to be overcome is the heterogeneity of EVs. All biofluids contain a complex mixture of EVs released by various mechanisms from various cell types. Cancer-derived EVs most likely constitute a small and variable fraction of EVs present in biofluids, therefore, cancer-derived molecules are highly diluted. Moreover, various subsets of EVs produced by the same cell type have been shown to differ in their protein and RNA composition [237–239]. Hence, a deeper understanding into the heterogeneity of EVs in terms of their biophysical properties, composition of surface molecules and molecular cargo, is needed to develop more specific and sensitive assays for detecting EV-based cancer biomarkers. Finally, when EVs began to be considered as a potential source for biomarkers, there was in general an incomplete understanding in the EV community about the specific clinical needs and the long and thorough pipeline required for the successful development of clinical biomarkers [233, 240–242]. These initial studies constitute, however, a proof-of-principle that can be further developed in multidisciplinary teams in the coming years. Importantly, in the last few years, EV-biomarker studies have been more carefully planned and have included more patients. Therefore, it is to be expected that in a close future some of these biomarkers will move from the discovery phase to analytical validation, clinical validation and finally clinical application. Besides, it would be very interesting to investigate the function of novel EV biomarkers in the disease and their possible use as therapeutic target.

**Table 4:** Challenges and possible solutions for the analysis of EVs in liquid biopsies for prostate cancer.

Limitations & challenges	Solutions & future directions
**Translational**	
Poor reproducibility due to incomplete description of patient cohorts and biofluid collection and storage protocols.	- Increase awareness of reporting importance. - Implement guidelines for minimal reporting information.
Low availability of biobanks designed specifically for the needs of EV research.	- Better understanding of how biofluid collection and storage parameters affect EV properties. - Establish biobanks that match the needs of EV research.
High variability of study outcome due to low cohort size and lack of cross-validation.	- Use larger cohorts. - Increase the number of multisite studies.
Biomarker studies do not always address a real clinical need in prostate cancer.	- Identify clinical questions where EVs analysis can be an advantage. - Improve dialog between EV scientists, urologists and oncologists.
Sub-optimal performance of the identified EV biomarkers.	- Use multiplexing of different types of EV molecules such as different RNA molecular types, or RNA and proteins. - Use multiplexing of EV molecules and non EV molecules in the biofluid. - Study if the candidate biomarker performs better in other biofluid or in a specific subpopulation of prostate-cancer patients. - Study EV molecules that have not received much attention so far and molecular modifications (e.g. lipids, glycans).
**Methodological**	
Poor reproducibility due to incomplete description of EV isolation methods.	- Increase awareness of reporting importance. - Implement guidelines for minimal reporting information. - Advocate transparent information sharing about the components of commercial kits for EV isolation.
Poor reproducibility due to the high variety of EV-isolation methods.	- Use reference materials to compare and normalise the results obtained by different methods. - Explore direct analysis of EVs without prior isolation.
Misinterpretation of results due to confounders in biofluids.	- Perform control experiments to confirm that the molecule of interest is associated with EVs. - Use spike-in and endogenous controls. - Register and monitor biofluid parameters (e.g. blood and uromodulin in urine, urine pH and protein concentration, haemolysis, platelets, lipoprotein content).
High heterogeneity of the EV population in biofluids (different release mechanisms, different cells of origin) and low relative abundance of prostate-derived EVs hamper the detection of prostate-cancer biomarkers.	- Gain insight into how different EV-isolation methods affect the yield of different EV populations. - Identify prostate and prostate-cancer-specific EV molecules. - Develop methods to isolate prostate-specific EV populations.
Low sensitivity of the analytical method.	- Develop more sensitive analytical tools for EV analysis. - Optimise yield of EV-isolation methods. - For urine, perform DRE to increase prostate-derived EV numbers.
Lack of optimal normalisation methods and endogenous normalisation controls.	- Design and execute systematic studies addressing normalisation methods and their optimal utilisation. - Develop reference materials.
Laboratory methodology is too complex for clinical implementation.	- Develop robust, fast and cheap methods for detection and quantification of EVs and EV biomarkers. - Improve communication between academia, hospitals and industry.

Today, it is considered that multiplex biomarker assays perform better than single cancer biomarkers, and many available cancer-diagnostic assays are based on the detection of several molecules [4, 6–8]. In this respect, EVs are particularly interesting because they contain hundreds of proteins, nucleic acids, lipids and metabolites. EV molecules belonging to the same molecular type can be analysed together, but different types of molecules such as proteins and RNAs can also be analysed in the same sample. This constitutes a promising approach, still in its early days [29]. Moreover, the molecular content of EVs could be analysed together with other liquid biomarkers to increase the robustness of cancer diagnostic tests.

## Conclusion

The implementation of novel liquid biopsies in the clinic is necessary to bring PCa care to the next level in the field of precision medicine. Body fluids are easily accessible, enabling screening of men at risk of developing PCa and real-time monitoring of disease progression and treatment responses. Therefore, liquid biopsies are expected to become part of PCa care from diagnosis till the end of treatment, helping to improve the treatment-response rate and reduce unnecessary side effects. To reach these goals, we need to continue the search for biomarkers addressing real clinical needs, to increase the number of prospective studies to show clinical benefits of the putative markers already known and to analyse the costs of using biomarkers in the clinic from a societal perspective.

While the majority of the identified EV-based biomarkers have still not reached the clinic, many studies have shown their clinical potential, and the first test has been commercialised [159, 206]. In the coming years, we expect to obtain a better understanding of (cancer) EV biology and develop more precise and sensitive technology for their detection. Furthermore, the use of a multidisciplinary approach in the design of EV-biomarker studies, the design of clinically friendly EV analytical assays and a good understanding of the requirements for regulatory approval will help to accelerate the translation of EV-based biomarkers into clinical assays for PCa and other diseases.

## Data Availability

Not applicable.

## References

[1] Sung H, Ferlay J, Siegel RL, Laversanne M, Soerjomataram I, Jemal A (2021). Global cancer statistics 2020: GLOBOCAN estimates of incidence and mortality worldwide for 36 cancers in 185 countries. CA Cancer J Clin.

[2] Egevad L, Delahunt B, Srigley JR, Samaratunga H (2016). International society of urological pathology (ISUP) grading of prostate cancer—An ISUP consensus on contemporary grading. APMIS.

[3] D’Amico AV, Whittington R, Malkowicz SB, Cote K, Loffredo M, Schultz D (2002). Biochemical outcome after radical prostatectomy or external beam radiation therapy for patients with clinically localized prostate carcinoma in the prostate specific antigen era. Cancer..

[4] Prensner JR, Rubin MA, Wei JT, Chinnaiyan AM (2012). Beyond PSA: the next generation of prostate cancer biomarkers. Sci Transl Med..

[5] De Angelis G, Rittenhouse HG, Mikolajczyk SD. Blair Shamel, L., Semjonow, A. Twenty years of PSA: from prostate antigen to tumor marker. Rev Urol. 2007;9:113–23. PMC2002501 17934568

[6] Murphy L, Prencipe M, Gallagher WM, Watson RW (2015). Commercialized biomarkers: new horizons in prostate cancer diagnostics. Expert Rev Mol Diagn..

[7] Tonry C, Finn S, Armstrong J, Pennington SR (2020). Clinical proteomics for prostate cancer: understanding prostate cancer pathology and protein biomarkers for improved disease management. Clin Proteom.

[8] Sharma, P, Zargar-Shoshtari, K, Pow-Sang, JM. Biomarkers for prostate cancer: present challenges and future opportunities. Futur Sci OA. 2016;2:FSO72. 10.4155/fso.15.72 PMC5137959 28031932

[9] Silberstein JL, Pal SK, Lewis B, Sartor O (2013). Current clinical challenges in prostate cancer. Transl Androl Urol.

[10] Gutman AB, Gutman EB (1938). An “acid“ phosphatase occurring in the serum of patients with metastasizing carcinoma of the prostate gland. J Clin Invest.

[11] Babayan A, Pantel K (2018). Advances in liquid biopsy approaches for early detection and monitoring of cancer. Genome Med.

[12] Pantel K, Alix-Panabieres C (2019). Liquid biopsy and minimal residual disease—latest advances and implications for cure. Nat Rev Clin Oncol.

[13] Siravegna G, Mussolin B, Venesio T, Marsoni S, Seoane J, Dive C (2019). How liquid biopsies can change clinical practice in oncology. Ann Oncol.

[14] Amintas, S, Bedel, A, Moreau-Gaudry, F, Boutin, J, Buscail, L, Merlio, JP et al. Circulating tumor cell clusters: united we stand divided we fall. Int J Mol Sci. 2020;21:2653. 10.3390/ijms21072653 PMC7177734 32290245

[15] Mout L, van Dessel LF, Kraan J, de Jong AC, Neves RPL, Erkens-Schulze S (2021). Generating human prostate cancer organoids from leukapheresis enriched circulating tumour cells. Eur J Cancer.

[16] Alix-Panabieres C, Pantel K (2021). Liquid biopsy: from discovery to clinical application. Cancer Disco.

[17] De Rubis G, Rajeev Krishnan S, Bebawy M (2019). Liquid biopsies in cancer diagnosis, monitoring, and prognosis. Trends Pharm Sci.

[18] Zapatero A, Gomez-Caamano A, Cabeza Rodriguez MA, Muinelo-Romay L, Martin de Vidales C, Abalo A (2020). Detection and dynamics of circulating tumor cells in patients with high-risk prostate cancer treated with radiotherapy and hormones: a prospective phase II study. Radiat Oncol.

[19] Meyer CP, Pantel K, Tennstedt P, Stroelin P, Schlomm T, Heinzer H (2016). Limited prognostic value of preoperative circulating tumor cells for early biochemical recurrence in patients with localized prostate cancer. Urol Oncol.

[20] Goldkorn A, Tangen C, Plets M, Morrison GJ, Cunha A, Xu T (2021). Baseline circulating tumor cell count as a prognostic marker of PSA response and disease progression in metastatic castrate-sensitive prostate cancer (SWOG S1216). Clin Cancer Res.

[21] Scher HI, Armstrong AJ, Schonhoft JD, Gill A, Zhao JL, Barnett E (2021). Development and validation of circulating tumour cell enumeration (Epic Sciences) as a prognostic biomarker in men with metastatic castration-resistant prostate cancer. Eur J Cancer.

[22] Wang C, Zhang Z, Chong W, Luo R, Myers RE, Gu J, et al. Improved prognostic stratification using circulating tumor cell clusters in patients with metastatic castration-resistant prostate cancer. Cancers. 2021;13:268. 10.3390/cancers13020268 PMC7828213 33450815

[23] Strati A, Zavridou M, Bournakis E, Mastoraki S, Lianidou E (2019). Expression pattern of androgen receptors, AR-V7 and AR-567es, in circulating tumor cells and paired plasma-derived extracellular vesicles in metastatic castration resistant prostate cancer. Analyst..

[24] Wan JCM, Massie C, Garcia-Corbacho J, Mouliere F, Brenton JD, Caldas C (2017). Liquid biopsies come of age: towards implementation of circulating tumour DNA. Nat Rev Cancer.

[25] Saarenheimo J, Eigeliene N, Andersen H, Tiirola M, Jekunen A (2019). The value of liquid biopsies for guiding therapy decisions in non-small cell lung cancer. Front Oncol.

[26] Leja M, Line A (2021). Early detection of gastric cancer beyond endoscopy—new methods. Best Pr Res Clin Gastroenterol.

[27] Diaz LA, Bardelli A (2014). Liquid biopsies: genotyping circulating tumor DNA. J Clin Oncol.

[28] Xie M, Lu C, Wang J, McLellan MD, Johnson KJ, Wendl MC (2014). Age-related mutations associated with clonal hematopoietic expansion and malignancies. Nat Med.

[29] Yu W, Hurley J, Roberts D, Chakrabortty SK, Enderle D, Noerholm M (2021). Exosome-based liquid biopsies in cancer: opportunities and challenges. Ann Oncol.

[30] Vasconcelos MH, Caires HR, Abols A, Xavier CPR, Line A (2019). Extracellular vesicles as a novel source of biomarkers in liquid biopsies for monitoring cancer progression and drug resistance. Drug Resist Updat.

[31] Thery C, Witwer KW, Aikawa E, Alcaraz MJ, Anderson JD, Andriantsitohaina R (2018). Minimal information for studies of extracellular vesicles 2018 (MISEV2018): a position statement of the International Society for Extracellular Vesicles and update of the MISEV2014 guidelines. J Extracell Vesicles.

[32] Yanez-Mo M, Siljander PR, Andreu Z, Zavec AB, Borras FE, Buzas EI (2015). Biological properties of extracellular vesicles and their physiological functions. J Extracell Vesicles.

[33] Hessvik NP, Llorente A (2018). Current knowledge on exosome biogenesis and release. Cell Mol Life Sci..

[34] van Niel G, D’Angelo G, Raposo G (2018). Shedding light on the cell biology of extracellular vesicles. Nat Rev Mol Cell Biol.

[35] Mathieu M, Martin-Jaular L, Lavieu G, Thery C (2019). Specificities of secretion and uptake of exosomes and other extracellular vesicles for cell-to-cell communication. Nat Cell Biol..

[36] Gurung S, Perocheau D, Touramanidou L, Baruteau J (2021). The exosome journey: from biogenesis to uptake and intracellular signalling. Cell Commun Signal.

[37] Caruso S, Poon IKH (2018). Apoptotic cell-derived extracellular vesicles: more than just debris. Front Immunol.

[38] Vagner T, Spinelli C, Minciacchi VR, Balaj L, Zandian M, Conley A (2018). Large extracellular vesicles carry most of the tumour DNA circulating in prostate cancer patient plasma. J Extracell Vesicles.

[39] Minciacchi VR, You S, Spinelli C, Morley S, Zandian M, Aspuria PJ (2015). Large oncosomes contain distinct protein cargo and represent a separate functional class of tumor-derived extracellular vesicles. Oncotarget..

[40] Murillo OD, Thistlethwaite W, Rozowsky J, Subramanian SL, Lucero R, Shah N (2019). exRNA atlas analysis reveals distinct extracellular RNA cargo types and their carriers present across human biofluids. Cell..

[41] Zonneveld MI, van Herwijnen MJC, Fernandez-Gutierrez MM, Giovanazzi A, de Groot AM, Kleinjan M (2021). Human milk extracellular vesicles target nodes in interconnected signalling pathways that enhance oral epithelial barrier function and dampen immune responses. J Extracell Vesicles.

[42] Johnstone RM, Adam M, Hammond JR, Orr L, Turbide C (1987). Vesicle formation during reticulocyte maturation. Association of plasma membrane activities with released vesicles (exosomes). J Biol Chem.

[43] Tkach M, Thery C (2016). Communication by extracellular vesicles: where we are and where we need to go. Cell..

[44] Henrich SE, McMahon KM, Plebanek MP, Calvert AE, Feliciano TJ, Parrish S (2020). Prostate cancer extracellular vesicles mediate intercellular communication with bone marrow cells and promote metastasis in a cholesterol-dependent manner. J Extracell Vesicles.

[45] Peinado H, Zhang H, Matei IR, Costa-Silva B, Hoshino A, Rodrigues G (2017). Pre-metastatic niches: organ-specific homes for metastases. Nat Rev Cancer.

[46] Williams C, Royo F, Aizpurua-Olaizola O, Pazos R, Boons GJ, Reichardt NC (2018). Glycosylation of extracellular vesicles: current knowledge, tools and clinical perspectives. J Extracell Vesicles.

[47] Colombo M, Raposo G, Thery C (2014). Biogenesis, secretion, and intercellular interactions of exosomes and other extracellular vesicles. Annu Rev Cell Dev Biol.

[48] O’Brien K, Breyne K, Ughetto S, Laurent LC, Breakefield XO (2020). RNA delivery by extracellular vesicles in mammalian cells and its applications. Nat Rev Mol Cell Biol.

[49] Peruzzotti-Jametti L, Bernstock JD, Willis CM, Manferrari G, Rogall R, Fernandez-Vizarra E (2021). Neural stem cells traffic functional mitochondria via extracellular vesicles. PLoS Biol.

[50] Skotland T, Sagini K, Sandvig K, Llorente A (2020). An emerging focus on lipids in extracellular vesicles. Adv Drug Deliv Rev.

[51] Kakarla R, Hur J, Kim YJ, Kim J, Chwae YJ (2020). Apoptotic cell-derived exosomes: messages from dying cells. Exp Mol Med.

[52] Al-Nedawi K, Meehan B, Micallef J, Lhotak V, May L, Guha A (2008). Intercellular transfer of the oncogenic receptor EGFRvIII by microvesicles derived from tumour cells. Nat Cell Biol.

[53] Peinado H, Aleckovic M, Lavotshkin S, Matei I, Costa-Silva B, Moreno-Bueno G (2012). Melanoma exosomes educate bone marrow progenitor cells toward a pro-metastatic phenotype through MET. Nat Med.

[54] Broggi MAS, Maillat L, Clement CC, Bordry N, Corthesy P, Auger A (2019). Tumor-associated factors are enriched in lymphatic exudate compared to plasma in metastatic melanoma patients. J Exp Med.

[55] Garcia-Silva S, Benito-Martin A, Sanchez-Redondo S, Hernandez-Barranco A, Ximenez-Embun P, Nogues L (2019). Use of extracellular vesicles from lymphatic drainage as surrogate markers of melanoma progression and BRAF (V600E) mutation. J Exp Med.

[56] Lazaro-Ibanez E, Sanz-Garcia A, Visakorpi T, Escobedo-Lucea C, Siljander P, Ayuso-Sacido A (2014). Different gDNA content in the subpopulations of prostate cancer extracellular vesicles: apoptotic bodies, microvesicles, and exosomes. Prostate..

[57] Mohrmann L, Huang HJ, Hong DS, Tsimberidou AM, Fu S, Piha-Paul SA (2018). Liquid biopsies using plasma exosomal nucleic acids and plasma cell-Free DNA compared with clinical outcomes of patients with advanced cancers. Clin Cancer Res.

[58] Wan Y, Liu B, Lei H, Zhang B, Wang Y, Huang H (2018). Nanoscale extracellular vesicle-derived DNA is superior to circulating cell-free DNA for mutation detection in early-stage non-small-cell lung cancer. Ann Oncol.

[59] Sahoo S, Adamiak M, Mathiyalagan P, Kenneweg F, Kafert-Kasting S, Thum T (2021). Therapeutic and diagnostic translation of extracellular vesicles in cardiovascular diseases: roadmap to the clinic. Circulation..

[60] Duijvesz D, Luider T, Bangma CH, Jenster G (2011). Exosomes as biomarker treasure chests for prostate cancer. Eur Urol..

[61] Minciacchi VR, Zijlstra A, Rubin MA, Di Vizio, D. Extracellular vesicles for liquid biopsy in prostate cancer: where are we and where are we headed? Prostate Cancer Prostatic Dis. 2017;20:251–8. 10.1038/pcan.2017.7 PMC5569339 28374743

[62] Urabe F, Kosaka N, Kimura T, Egawa S, Ochiya T (2018). Extracellular vesicles: toward a clinical application in urological cancer treatment. Int J Urol.

[63] Dhondt B, Van Deun J, Vermaerke S, de Marco A, Lumen N, De Wever O (2018). Urinary extracellular vesicle biomarkers in urological cancers: From discovery towards clinical implementation. Int J Biochem Cell Biol.

[64] Rimmer MP, Gregory CD, Mitchell RT (2021). Extracellular vesicles in urological malignancies. Biochim Biophys Acta Rev Cancer.

[65] Linxweiler J, Junker K (2020). Extracellular vesicles in urological malignancies: an update. Nat Rev Urol.

[66] Gao Z, Pang B, Li J, Gao N, Fan T, Li Y (2021). Emerging role of exosomes in liquid biopsy for monitoring prostate cancer invasion and metastasis. Front Cell Dev Biol.

[67] Hatano K, Fujita K (2021). Extracellular vesicles in prostate cancer: a narrative review. Transl Androl Urol.

[68] Campos-Fernandez E, Barcelos LS, de Souza AG, Goulart LR, Alonso-Goulart V (2019). Research landscape of liquid biopsies in prostate cancer. Am J Cancer Res.

[69] Vlaeminck-Guillem V (2018). Extracellular vesicles in prostate cancer carcinogenesis, diagnosis, and management. Front Oncol.

[70] Mitchell PJ, Welton J, Staffurth J, Court J, Mason MD, Tabi Z (2009). Can urinary exosomes act as treatment response markers in prostate cancer?. J Transl Med.

[71] Nilsson J, Skog J, Nordstrand A, Baranov V, Mincheva-Nilsson L, Breakefield XO (2009). Prostate cancer-derived urine exosomes: a novel approach to biomarkers for prostate cancer. Br J Cancer..

[72] Drake RR, Kislinger T (2014). The proteomics of prostate cancer exosomes. Expert Rev Proteom..

[73] Verze P, Cai T, Lorenzetti S (2016). The role of the prostate in male fertility, health and disease. Nat Rev Urol.

[74] Brody I, Ronquist G, Gottfries A (1983). Ultrastructural localization of the prostasome - an organelle in human seminal plasma. Ups J Med Sci..

[75] Ronquist G, Brody I, Gottfries A, Stegmayr B (1978). An Mg^2+^ and Ca^2+^-stimulated adenosine triphosphatase in human prostatic fluid: part I. Andrology..

[76] Ronquist G, Brody I (1985). The prostasome: its secretion and function in man. Biochim Biophys Acta..

[77] Ronquist G, Nilsson BO (2004). The Janus-faced nature of prostasomes: their pluripotency favours the normal reproductive process and malignant prostate growth. Prostate Cancer Prostatic Dis.

[78] Drake RR, Elschenbroich S, Lopez-Perez O, Kim Y, Ignatchenko V, Ignatchenko A (2010). In-depth proteomic analyses of direct expressed prostatic secretions. J Proteome Res.

[79] Baskaran S, Panner Selvam, MK, Agarwal, A. Exosomes of male reproduction. Adv Clin Chem. 2020;95:149–63. 10.1016/bs.acc.2019.08.004 32122522

[80] Zaichick V (2014). The prostatic urethra as a Venturi effect urine-jet pump to drain prostatic fluid. Med Hypotheses.

[81] Duijvesz D, Versluis CY, van der Fels CA, Vredenbregt-van den Berg MS, Leivo J, Peltola MT (2015). Immuno-based detection of extracellular vesicles in urine as diagnostic marker for prostate cancer. Int J Cancer.

[82] Hendriks RJ, Dijkstra S, Jannink SA, Steffens MG, van Oort IM, Mulders PF (2016). Comparative analysis of prostate cancer specific biomarkers PCA3 and ERG in whole urine, urinary sediments and exosomes. Clin Chem Lab Med.

[83] Erdbrugger U, Blijdorp CJ, Bijnsdorp IV, Borras FE, Burger D, Bussolati B (2021). Urinary extracellular vesicles: a position paper by the urine task force of the International Society for Extracellular Vesicles. J Extracell Vesicles.

[84] Hiemstra TF, Charles PD, Gracia T, Hester SS, Gatto L, Al-Lamki R (2014). Human urinary exosomes as innate immune effectors. J Am Soc Nephrol.

[85] Nolte-‘t Hoen E, Cremer T, Gallo RC, Margolis LB (2016). Extracellular vesicles and viruses: are they close relatives?. Proc Natl Acad Sci Usa.

[86] van Dongen HM, Masoumi N, Witwer KW, Pegtel DM (2016). Extracellular vesicles exploit viral entry routes for cargo delivery. Microbiol Mol Biol Rev.

[87] Goetsch, HE, Zhao, L, Gnegy, M, Imperiale, MJ, Love, NG, Wigginton, KR. Fate of the urinary tract virus BK human polyomavirus in source-separated urine. Appl Environ Microbiol. 2018;84:e02374-17. 10.1128/AEM.02374-17 PMC5861842 29374036

[88] Polo C, Perez JL, Mielnichuck A, Fedele CG, Niubo J, Tenorio A (2004). Prevalence and patterns of polyomavirus urinary excretion in immunocompetent adults and children. Clin Microbiol Infect.

[89] Lee Y, Park JY, Lee EH, Yang J, Jeong BR, Kim YK (2017). Rapid assessment of microbiota changes in individuals with autism spectrum disorder using bacteria-derived membrane vesicles in urine. Exp Neurobiol.

[90] Yoo JY, Rho M, You YA, Kwon EJ, Kim MH, Kym S (2016). 16S rRNA gene-based metagenomic analysis reveals differences in bacteria-derived extracellular vesicles in the urine of pregnant and non-pregnant women. Exp Mol Med.

[91] Principe S, Jones EE, Kim Y, Sinha A, Nyalwidhe JO, Brooks J (2013). In-depth proteomic analyses of exosomes isolated from expressed prostatic secretions in urine. Proteomics..

[92] Wang Z, Hill S, Luther JM, Hachey DL, Schey KL (2012). Proteomic analysis of urine exosomes by multidimensional protein identification technology (MudPIT). Proteomics..

[93] Øverbye A, Skotland T, Koehler CJ, Thiede B, Seierstad T, Berge V (2015). Identification of prostate cancer biomarkers in urinary exosomes. Oncotarget..

[94] Tripisciano C, Weiss R, Karuthedom George S, Fischer MB, Weber V (2020). Extracellular vesicles derived from platelets, red blood cells, and monocyte-like cells differ regarding their ability to induce factor XII-dependent thrombin generation. Front Cell Dev Biol.

[95] Stenman UH, Leinonen J, Zhang WM, Finne P (1999). Prostate-specific antigen. Semin Cancer Biol.

[96] Clayton, A, Buschmann, D, Byrd, JB, Carter, DRF, Cheng, L, Compton, C et al. Summary of the ISEV workshop on extracellular vesicles as disease biomarkers, held in Birmingham, UK, during December 2017. J Extracell Vesicles. 2018;7:1473707. 10.1080/20013078.2018.1473707 PMC5965025 31162490

[97] Clayton A, Boilard E, Buzás EI, Cheng L, Falcón-Perez JM, Gardiner C (2019). Considerations towards a roadmap for collection, handling and storage of blood extracellular vesicles. J Extracell Vesicles.

[98] Coumans FAW, Brisson AR, Buzas EI, Dignat-George F, Drees EEE, El-Andaloussi S (2017). Methodological Guidelines to Study Extracellular Vesicles. Circ Res.

[99] Witwer KW, Buzas EI, Bemis LT, Bora A, Lasser C, Lotvall J, et al. Standardization of sample collection, isolation and analysis methods in extracellular vesicle research. J Extracell Vesicles. 2013;2. 10.3402/jev.v2i0.20360 PMC3760646 24009894

[100] Lehmann S, Guadagni F, Moore H, Ashton G, Barnes M, Benson E (2012). Standard preanalytical coding for biospecimens: review and implementation of the Sample PREanalytical Code (SPREC). Biopreserv Biobank.

[101] Betsou F, Bilbao R, Case J, Chuaqui R, Clements JA, De Souza Y (2018). Standard PREanalytical code version 3.0. Biopreserv Biobank.

[102] Lacroix R, Judicone C, Poncelet P, Robert S, Arnaud L, Sampol J (2012). Impact of pre-analytical parameters on the measurement of circulating microparticles: towards standardization of protocol. J Thromb Haemost.

[103] Mullier F, Bailly N, Chatelain C, Chatelain B, Dogne JM (2013). Pre-analytical issues in the measurement of circulating microparticles: current recommendations and pending questions. J Thromb Haemost.

[104] Gyorgy B, Paloczi K, Kovacs A, Barabas E, Beko G, Varnai K (2014). Improved circulating microparticle analysis in acid-citrate dextrose (ACD) anticoagulant tube. Thromb Res.

[105] Tegegn TZ, De Paoli SH, Orecna M, Elhelu OK, Woodle SA, Tarandovskiy ID (2016). Characterization of procoagulant extracellular vesicles and platelet membrane disintegration in DMSO-cryopreserved platelets. J Extracell Vesicles.

[106] Rikkert LG, Coumans FAW, Hau CM, Terstappen L, Nieuwland R (2021). Platelet removal by single-step centrifugation. Platelets.

[107] Liangsupree T, Multia E, Riekkola ML (2021). Modern isolation and separation techniques for extracellular vesicles. J Chromatogr A.

[108] Karimi N, Cvjetkovic A, Jang SC, Crescitelli R, Hosseinpour Feizi, M.A., Nieuwland, R. et al. Detailed analysis of the plasma extracellular vesicle proteome after separation from lipoproteins. Cell Mol Life Sci. 2018;75:2873–86. 10.1007/s00018-018-2773-4 PMC6021463 29441425

[109] Fernandez-Llama P, Khositseth S, Gonzales PA, Star RA, Pisitkun T, Knepper MA (2010). Tamm-Horsfall protein and urinary exosome isolation. Kidney Int.

[110] Musante, L, Saraswat, M, Duriez, E, Byrne, B, Ravida, A, Domon, B et al. Biochemical and physical characterisation of urinary nanovesicles following CHAPS treatment. PLoS ONE. 2012;7:e37279. 10.1371/journal.pone.0037279 PMC3395701 22808001

[111] Xu X, Barreiro K, Musante L, Kretz O, Lin H, Zou H (2019). Management of Tamm-Horsfall protein for reliable urinary analytics. Proteom Clin Appl.

[112] Thery, C, Amigorena, S, Raposo, G, Clayton, A. Isolation and characterization of exosomes from cell culture supernatants and biological fluids. Curr Protoc Cell Biol. 2006;Chapter 3:Unit 3.22. 10.1002/0471143030.cb0322s30 18228490

[113] Gardiner C, Di Vizio D, Sahoo S, Thery C, Witwer KW, Wauben M (2016). Techniques used for the isolation and characterization of extracellular vesicles: results of a worldwide survey. J Extracell Vesicles.

[114] Konoshenko MY, Lekchnov EA, Vlassov AV, Laktionov PP (2018). Isolation of extracellular vesicles: general methodologies and latest trends. BioMed Res Int.

[115] Li P, Kaslan M, Lee SH, Yao J, Gao Z (2017). Progress in exosome isolation techniques. Theranostics..

[116] Royo, F, Thery, C, Falcon-Perez, JM, Nieuwland, R, Witwer, KW. Methods for separation and characterization of extracellular vesicles: results of a worldwide survey performed by the ISEV Rigor and Standardization subcommittee. Cells. 2020;9:1955. 10.3390/cells9091955 PMC7563174 32854228

[117] Gandham S, Su X, Wood J, Nocera AL, Alli SC, Milane L (2020). Technologies and standardization in research on extracellular vesicles. Trends Biotechnol.

[118] Choi JY, Kim S, Kwak HB, Park DH, Park JH, Ryu JS (2017). Extracellular vesicles as a source of urological biomarkers: lessons learned from advances and challenges in clinical applications to major diseases. Int Neurourol J.

[119] Ayala-Mar S, Donoso-Quezada J, Gallo-Villanueva RC, Perez-Gonzalez VH, Gonzalez-Valdez J (2019). Recent advances and challenges in the recovery and purification of cellular exosomes. Electrophoresis..

[120] Yamamoto T, Kosaka N, Ochiya T (2019). Latest advances in extracellular vesicles: from bench to bedside. Sci Technol Adv Mater.

[121] Van Deun J, Mestdagh P, Sormunen R, Cocquyt V, Vermaelen K, Vandesompele J, et al. The impact of disparate isolation methods for extracellular vesicles on downstream RNA profiling. J Extracell Vesicles. 2014;3. 10.3402/jev.v3.24858 PMC4169610 25317274

[122] Dhondt B, Geeurickx E, Tulkens J, Van Deun J, Vergauwen G, Lippens L (2020). Unravelling the proteomic landscape of extracellular vesicles in prostate cancer by density-based fractionation of urine. J Extracell Vesicles.

[123] Royo F, Zuniga-Garcia P, Sanchez-Mosquera P, Egia A, Perez A, Loizaga A (2016). Different EV enrichment methods suitable for clinical settings yield different subpopulations of urinary extracellular vesicles from human samples. J Extracell Vesicles.

[124] Macias M, Rebmann V, Mateos B, Varo N, Perez-Gracia JL, Alegre E (2019). Comparison of six commercial serum exosome isolation methods suitable for clinical laboratories. Effect in cytokine analysis. Clin Chem Lab Med.

[125] Blijdorp CJ, Tutakhel OAZ, Hartjes TA, van den Bosch TPP, van Heugten MH, Rigalli JP (2021). Comparing Approaches to Normalize, Quantify, and Characterize Urinary Extracellular Vesicles. J Am Soc Nephrol.

[126] Mateescu B, Kowal EJ, van Balkom BW, Bartel S, Bhattacharyya SN, Buzas EI (2017). Obstacles and opportunities in the functional analysis of extracellular vesicle RNA—an ISEV position paper. J Extracell Vesicles.

[127] Ye LF, He S, Wu X, Jiang S, Zhang RC, Yang ZS (2020). Detection of prostate cancer antigen 3 and prostate cancer susceptibility candidate in non-DRE urine improves diagnosis of prostate cancer in chinese population. Prostate Cancer..

[128] Danarto R, Astuti I, Umbas R, Haryana SM (2020). Urine miR-21-5p and miR-200c-3p as potential non-invasive biomarkers in patients with prostate cancer. Turk J Urol.

[129] Li Z, Li LX, Diao YJ, Wang J, Ye Y, Hao XK (2021). Identification of urinary exosomal miRNAs for the non-invasive diagnosis of prostate cancer. Cancer Manag Res.

[130] Vandesompele, J, De Preter, K, Pattyn, F, Poppe, B, Van Roy, N, De Paepe, A et al. Accurate normalization of real-time quantitative RT-PCR data by geometric averaging of multiple internal control genes. Genome Biol.2002;3, RESEARCH0034. 10.1186/gb-2002-3-7-research0034 PMC126239 12184808

[131] Srinivasan S, Duval MX, Kaimal V, Cuff C, Clarke SH (2019). Assessment of methods for serum extracellular vesicle small RNA sequencing to support biomarker development. J Extracell Vesicles.

[132] Liang Y, Lehrich BM, Zheng S, Lu M (2021). Emerging methods in biomarker identification for extracellular vesicle-based liquid biopsy. J Extracell Vesicles.

[133] Di Santo R, Romanò S, Mazzini A, Jovanovic S, Nocca G, Campi G (2021). Recent advances in the label-free characterization of exosomes for cancer liquid biopsy: from scattering and spectroscopy to nanoindentation and nanodevices. Nanomaterials.

[134] Martin-Gracia B, Martin-Barreiro A, Cuestas-Ayllon C, Grazu V, Line A, Llorente A (2020). Nanoparticle-based biosensors for detection of extracellular vesicles in liquid biopsies. J Mater Chem B.

[135] Lin S, Yu Z, Chen D, Wang Z, Miao J, Li Q (2020). Progress in microfluidics-based exosome separation and detection technologies for diagnostic applications. Small..

[136] Zhang P, Zhou X, He M, Shang Y, Tetlow AL, Godwin AK (2019). Ultrasensitive detection of circulating exosomes with a 3D-nanopatterned microfluidic chip. Nat Biomed Eng.

[137] Zhang P, He M, Zeng Y (2016). Ultrasensitive microfluidic analysis of circulating exosomes using a nanostructured graphene oxide/polydopamine coating. Lab Chip.

[138] Yang B, Chen Y, Shi J (2019). Exosome biochemistry and advanced nanotechnology for next-generation theranostic platforms. Adv Mater.

[139] Guo SC, Tao SC, Dawn H (2018). Microfluidics-based on-a-chip systems for isolating and analysing extracellular vesicles. J Extracell Vesicles.

[140] Yasui T, Yanagida T, Ito S, Konakade Y, Takeshita D, Naganawa T (2017). Unveiling massive numbers of cancer-related urinary-microRNA candidates via nanowires. Sci Adv.

[141] Tauro BJ, Greening DW, Mathias RA, Mathivanan S, Ji H, Simpson RJ (2013). Two distinct populations of exosomes are released from LIM1863 colon carcinoma cell-derived organoids. Mol Cell Proteom.

[142] Wunsch BH, Smith JT, Gifford SM, Wang C, Brink M, Bruce RL (2016). Nanoscale lateral displacement arrays for the separation of exosomes and colloids down to 20 nm. Nat Nanotechnol.

[143] Smith JT, Wunsch BH, Dogra N, Ahsen ME, Lee K, Yadav KK (2018). Integrated nanoscale deterministic lateral displacement arrays for separation of extracellular vesicles from clinically-relevant volumes of biological samples. Lab Chip.

[144] Ku A, Fredsoe J, Sorensen KD, Borre M, Evander M, Laurell T (2021). High-throughput and automated acoustic trapping of extracellular vesicles to identify microRNAs with diagnostic potential for prostate cancer. Front Oncol.

[145] Liu C, Guo J, Tian F, Yang N, Yan F, Ding Y (2017). Field-free isolation of exosomes from extracellular vesicles by microfluidic viscoelastic flows. ACS Nano.

[146] Asghari M, Cao X, Mateescu B, van Leeuwen D, Aslan MK, Stavrakis S (2020). Oscillatory viscoelastic microfluidics for efficient focusing and separation of nanoscale species. ACS Nano.

[147] Ziaei P, Berkman CE, Norton MG (2018). Isolation and detection of tumor-derived extracellular vesicles. ACS Appl Nano Mater.

[148] Kanwar SS, Dunlay CJ, Simeone DM, Nagrath S (2014). Microfluidic device (ExoChip) for on-chip isolation, quantification and characterization of circulating exosomes. Lab Chip.

[149] Zhao Z, Yang Y, Zeng Y, He M (2016). A microfluidic ExoSearch chip for multiplexed exosome detection towards blood-based ovarian cancer diagnosis. Lab Chip.

[150] Jeong S, Park J, Pathania D, Castro CM, Weissleder R, Lee H (2016). Integrated magneto-electrochemical sensor for exosome analysis. ACS Nano.

[151] Hernandez-Neuta I, Neumann F, Brightmeyer J, Ba Tis T, Madaboosi N, Wei Q (2019). Smartphone-based clinical diagnostics: towards democratization of evidence-based health care. J Intern Med.

[152] Ko J, Hemphill MA, Gabrieli D, Wu L, Yelleswarapu V, Lawrence G (2016). Smartphone-enabled optofluidic exosome diagnostic for concussion recovery. Sci Rep..

[153] Liang LG, Kong MQ, Zhou S, Sheng YF, Wang P, Yu T (2017). An integrated double-filtration microfluidic device for isolation, enrichment and quantification of urinary extracellular vesicles for detection of bladder cancer. Sci Rep..

[154] Califf RM (2018). Biomarker definitions and their applications. Exp Biol Med..

[155] Motamedinia P, Scott AN, Bate KL, Sadeghi N, Salazar G, Shapiro E (2016). Urine exosomes for non-invasive assessment of gene expression and mutations of prostate cancer. PLoS ONE.

[156] Dijkstra S, Birker IL, Smit FP, Leyten GH, de Reijke TM, van Oort IM (2014). Prostate cancer biomarker profiles in urinary sediments and exosomes. J Urol.

[157] Donovan MJ, Noerholm M, Bentink S, Belzer S, Skog J, O’Neill V (2015). A molecular signature of PCA3 and ERG exosomal RNA from non-DRE urine is predictive of initial prostate biopsy result. Prostate Cancer Prostatic Dis.

[158] McKiernan J, Donovan MJ, O’Neill V, Bentink S, Noerholm M, Belzer S (2016). A novel urine exosome gene expression assay to predict high-grade prostate cancer at initial biopsy. JAMA Oncol.

[159] McKiernan J, Donovan MJ, Margolis E, Partin A, Carter B, Brown G (2018). A prospective adaptive utility trial to validate performance of a novel urine exosome gene expression assay to predict high-grade prostate cancer in patients with prostate-specific antigen 2–10 ng/ml at initial biopsy. Eur Urol.

[160] Tutrone R, Donovan MJ, Torkler P, Tadigotla V, McLain T, Noerholm M (2020). Clinical utility of the exosome based ExoDx Prostate(IntelliScore) EPI test in men presenting for initial Biopsy with a PSA 2-10 ng/mL. Prostate Cancer Prostatic Dis.

[161] Woo J, Santasusagna S, Banks J, Pastor-Lopez S, Yadav K, Carceles-Cordon M (2020). Urine extracellular vesicle GATA2 mRNA discriminates biopsy result in men with suspicion of prostate cancer. J Urol.

[162] Kohaar I, Chen Y, Banerjee S, Borbiev T, Kuo HC, Ali A (2021). A urine exosome gene expression panel distinguishes between indolent and aggressive prostate cancers at biopsy. J Urol.

[163] Royo F, Zuniga-Garcia P, Torrano V, Loizaga A, Sanchez-Mosquera P, Ugalde-Olano A (2016). Transcriptomic profiling of urine extracellular vesicles reveals alterations of CDH3 in prostate cancer. Oncotarget..

[164] Neeb A, Hefele S, Bormann S, Parson W, Adams F, Wolf P (2014). Splice variant transcripts of the anterior gradient 2 gene as a marker of prostate cancer. Oncotarget..

[165] Yaman Agaoglu F, Kovancilar M, Dizdar Y, Darendeliler E, Holdenrieder S, Dalay N (2011). Investigation of miR-21, miR-141, and miR-221 in blood circulation of patients with prostate cancer. Tumour Biol.

[166] Zhang HL, Yang LF, Zhu Y, Yao XD, Zhang SL, Dai B (2011). Serum miRNA-21: elevated levels in patients with metastatic hormone-refractory prostate cancer and potential predictive factor for the efficacy of docetaxel-based chemotherapy. Prostate..

[167] Li M, Rai AJ, DeCastro GJ, Zeringer E, Barta T, Magdaleno S (2015). An optimized procedure for exosome isolation and analysis using serum samples: Application to cancer biomarker discovery. Methods..

[168] Endzelins E, Berger A, Melne V, Bajo-Santos C, Sobolevska K, Abols A (2017). Detection of circulating miRNAs: comparative analysis of extracellular vesicle-incorporated miRNAs and cell-free miRNAs in whole plasma of prostate cancer patients. BMC Cancer.

[169] Samsonov R, Shtam T, Burdakov V, Glotov A, Tsyrlina E, Berstein L (2016). Lectin-induced agglutination method of urinary exosomes isolation followed by mi-RNA analysis: application for prostate cancer diagnostic. Prostate..

[170] Foj L, Ferrer F, Serra M, Arevalo A, Gavagnach M, Gimenez N (2017). Exosomal and non-exosomal urinary miRNAs in prostate cancer detection and prognosis. Prostate..

[171] Haldrup C, Kosaka N, Ochiya T, Borre M, Hoyer S, Orntoft TF (2014). Profiling of circulating microRNAs for prostate cancer biomarker discovery. Drug Deliv Transl Res.

[172] Mitchell PS, Parkin RK, Kroh EM, Fritz BR, Wyman SK, Pogosova-Agadjanyan EL (2008). Circulating microRNAs as stable blood-based markers for cancer detection. Proc Natl Acad Sci USA.

[173] Koppers-Lalic D, Hackenberg M, de Menezes R, Misovic B, Wachalska M, Geldof A (2016). Noninvasive prostate cancer detection by measuring miRNA variants (isomiRs) in urine extracellular vesicles. Oncotarget..

[174] Li Z, Ma YY, Wang J, Zeng XF, Li R, Kang W (2016). Exosomal microRNA-141 is upregulated in the serum of prostate cancer patients. Onco Targets Ther.

[175] Li W, Dong Y, Wang KJ, Deng Z, Zhang W, Shen HF (2020). Plasma exosomal miR-125a-5p and miR-141-5p as non-invasive biomarkers for prostate cancer. Neoplasma..

[176] Xu Y, Qin S, An T, Tang Y, Huang Y, Zheng L (2017). MiR-145 detection in urinary extracellular vesicles increase diagnostic efficiency of prostate cancer based on hydrostatic filtration dialysis method. Prostate..

[177] Wani S, Kaul D, Mavuduru RS, Kakkar N, Bhatia A (2017). Urinary-exosomal miR-2909: a novel pathognomonic trait of prostate cancer severity. J Biotechnol.

[178] Bhagirath D, Yang TL, Bucay N, Sekhon K, Majid S, Shahryari V (2018). microRNA-1246 Is an exosomal biomarker for aggressive prostate cancer. Cancer Res.

[179] Bryant RJ, Pawlowski T, Catto JW, Marsden G, Vessella RL, Rhees B (2012). Changes in circulating microRNA levels associated with prostate cancer. Br J Cancer.

[180] Rodriguez M, Bajo-Santos C, Hessvik NP, Lorenz S, Fromm B, Berge V (2017). Identification of non-invasive miRNAs biomarkers for prostate cancer by deep sequencing analysis of urinary exosomes. Mol Cancer.

[181] Matsuzaki K, Fujita K, Tomiyama E, Hatano K, Hayashi Y, Wang C (2021). MiR-30b-3p and miR-126-3p of urinary extracellular vesicles could be new biomarkers for prostate cancer. Transl Androl Urol.

[182] Zhou C, Chen Y, He X, Zheng Z, Xue D (2020). Functional implication of exosomal miR-217 and miR-23b-3p in the progression of prostate cancer. Onco Targets Ther.

[183] Barcelo M, Castells M, Bassas L, Vigues F, Larriba S (2019). Semen miRNAs contained in exosomes as non-invasive biomarkers for prostate cancer diagnosis. Sci Rep..

[184] Mercadal M, Herrero C, Lopez-Rodrigo O, Castells M, de la Fuente A, Vigues F, et al. Impact of extracellular vesicle isolation methods on downstream mirna analysis in semen: a comparative study. Int J Mol Sci. 2020;21:5949. 10.3390/ijms21175949 PMC7504614 32824915

[185] Logozzi M, Angelini DF, Iessi E, Mizzoni D, Di Raimo R, Federici C (2017). Increased PSA expression on prostate cancer exosomes in in vitro condition and in cancer patients. Cancer Lett.

[186] Logozzi M, Angelini DF, Giuliani A, Mizzoni D, Di Raimo R, Maggi M, et al. Increased plasmatic levels of PSA-expressing exosomes distinguish prostate cancer patients from benign prostatic hyperplasia: a prospective study. Cancers. 2019;11:1449. 10.3390/cancers11101449 PMC6826376 31569672

[187] Sequeiros T, Rigau M, Chiva C, Montes M, Garcia-Grau I, Garcia M (2017). Targeted proteomics in urinary extracellular vesicles identifies biomarkers for diagnosis and prognosis of prostate cancer. Oncotarget..

[188] Soekmadji C, Riches JD, Russell PJ, Ruelcke JE, McPherson S, Wang C (2017). Modulation of paracrine signaling by CD9 positive small extracellular vesicles mediates cellular growth of androgen deprived prostate cancer. Oncotarget..

[189] Carlsson L, Nilsson O, Larsson A, Stridsberg M, Sahlen G, Ronquist G (2003). Characteristics of human prostasomes isolated from three different sources. Prostate..

[190] Tavoosidana G, Ronquist G, Darmanis S, Yan J, Carlsson L, Wu D (2011). Multiple recognition assay reveals prostasomes as promising plasma biomarkers for prostate cancer. Proc Natl Acad Sci USA.

[191] Khan S, Jutzy JM, Valenzuela MM, Turay D, Aspe JR, Ashok A (2012). Plasma-derived exosomal survivin, a plausible biomarker for early detection of prostate cancer. PLoS ONE.

[192] Khan S, Simpson J, Lynch JC, Turay D, Mirshahidi S, Gonda A (2017). Racial differences in the expression of inhibitors of apoptosis (IAP) proteins in extracellular vesicles (EV) from prostate cancer patients. PLoS ONE.

[193] Fujita K, Kume H, Matsuzaki K, Kawashima A, Ujike T, Nagahara A (2017). Proteomic analysis of urinary extracellular vesicles from high Gleason score prostate cancer. Sci Rep..

[194] Gabriel K, Ingram A, Austin R, Kapoor A, Tang D, Majeed F (2013). Regulation of the tumor suppressor PTEN through exosomes: a diagnostic potential for prostate cancer. PLoS ONE.

[195] Wang L, Skotland T, Berge V, Sandvig K, Llorente A (2017). Exosomal proteins as prostate cancer biomarkers in urine: From mass spectrometry discovery to immunoassay-based validation. Eur J Pharm Sci.

[196] Li S, Zhao Y, Chen W, Yin L, Zhu J, Zhang H (2018). Exosomal ephrinA2 derived from serum as a potential biomarker for prostate cancer. J Cancer.

[197] Chung JW, Kim HT, Ha YS, Lee EH, Chun SY, Lee CH (2021). Identification of a novel non-invasive biological marker to overcome the shortcomings of PSA in diagnosis and risk stratification for prostate cancer: Initial prospective study of developmental endothelial locus-1 protein. PLoS ONE.

[198] Bijnsdorp IV, Geldof AA, Lavaei M, Piersma SR, van Moorselaar RJ, Jimenez CR. Exosomal ITGA3 interferes with non-cancerous prostate cell functions and is increased in urine exosomes of metastatic prostate cancer patients. J Extracell Vesicles. 2013;2. 10.3402/jev.v2i0.22097 PMC3873120 24371517

[199] Kawakami K, Fujita Y, Matsuda Y, Arai T, Horie K, Kameyama K (2017). Gamma-glutamyltransferase activity in exosomes as a potential marker for prostate cancer. BMC Cancer.

[200] Khanna, K, Salmond, N, Lynn, KS, Leong, HS, Williams, KC. Clinical significance of STEAP1 extracellular vesicles in prostate cancer. Prostate Cancer Prostatic Dis. 2021;24:802–811. 10.1038/s41391-021-00319-2 PMC8384631 33589770

[201] Skotland T, Ekroos K, Kauhanen D, Simolin H, Seierstad T, Berge V (2017). Molecular lipid species in urinary exosomes as potential prostate cancer biomarkers. Eur J Cancer.

[202] Clos-Garcia M, Loizaga-Iriarte A, Zuniga-Garcia P, Sanchez-Mosquera P, Rosa Cortazar A, Gonzalez E (2018). Metabolic alterations in urine extracellular vesicles are associated to prostate cancer pathogenesis and progression. J Extracell Vesicles..

[203] Puhka M, Takatalo M, Nordberg M-E, Valkonen S, Nandania J, Aatonen M (2017). Metabolomic profiling of extracellular vesicles and alternative normalization methods reveal enriched metabolites and strategies to study prostate cancer-related changes. Theranostics..

[204] Isin M, Uysaler E, Ozgur E, Koseoglu H, Sanli O, Yucel OB (2015). Exosomal lncRNA-p21 levels may help to distinguish prostate cancer from benign disease. Front Genet.

[205] Wang YH, Ji J, Wang BC, Chen H, Yang ZH, Wang K (2018). Tumor-derived exosomal long noncoding RNAs as promising diagnostic biomarkers for prostate cancer. Cell Physiol Biochem.

[206] Wang WW, Sorokin I, Aleksic I, Fisher H, Kaufman RP, Winer A (2020). Expression of small noncoding RNAs in urinary exosomes classifies prostate cancer into indolent and aggressive disease. J Urol.

[207] Krafft C, Wilhelm K, Eremin A, Nestel S, von Bubnoff N, Schultze-Seemann W (2017). A specific spectral signature of serum and plasma-derived extracellular vesicles for cancer screening. Nanomedicine..

[208] Wang Y, Li Q, Shi H, Tang K, Qiao L, Yu G (2020). Microfluidic Raman biochip detection of exosomes: a promising tool for prostate cancer diagnosis. Lab Chip.

[209] Huang X, Yuan T, Liang M, Du M, Xia S, Dittmar R (2015). Exosomal miR-1290 and miR-375 as prognostic markers in castration-resistant prostate cancer. Eur Urol.

[210] Zavridou M, Strati A, Bournakis E, Smilkou S, Tserpeli V, Lianidou E (2021). Prognostic significance of gene expression and DNA methylation markers in circulating tumor cells and paired plasma derived exosomes in metastatic castration resistant prostate cancer. Cancers..

[211] Biggs, CN, Siddiqui, KM, Al-Zahrani, AA, Pardhan, S, Brett, SI, Guo QQ, et al. Prostate extracellular vesicles in patient plasma as a liquid biopsy platform for prostate cancer using nanoscale flow cytometry. Oncotarget. 2016;78839–49. 10.18632/oncotarget.6983 PMC4891008 26814433

[212] Nanou A, Coumans FAW, van Dalum G, Zeune LL, Dolling D, Onstenk W (2018). Circulating tumor cells, tumor-derived extracellular vesicles and plasma cytokeratins in castration-resistant prostate cancer patients. Oncotarget..

[213] Nanou A, Miller MC, Zeune LL, de Wit S, Punt CJA, Groen HJM (2020). Tumour-derived extracellular vesicles in blood of metastatic cancer patients associate with overall survival. Br J Cancer.

[214] Connell SP, Yazbek-Hanna M, McCarthy F, Hurst R, Webb M, Curley H (2019). A four-group urine risk classifier for predicting outcomes in patients with prostate cancer. BJU Int.

[215] Fredsøe J, Rasmussen AKI, Mouritzen P, Borre M, Ørntoft T, Sørensen KD (2019). A five-microRNA model (pCaP) for predicting prostate cancer aggressiveness using cell-free urine. Int J Cancer..

[216] Tian S, Lei Z, Gong Z, Sun Z, Xu D, Piao M (2020). Clinical implication of prognostic and predictive biomarkers for castration-resistant prostate cancer: a systematic review. Cancer Cell Int.

[217] Rice MA, Stoyanova, T. Biomarkers for diagnosis and prognosis of prostate cancer. Prostatectomy. 2019;9.

[218] Del Re M, Crucitta S, Sbrana A, Rofi E, Paolieri F, Gianfilippo G (2019). Androgen receptor (AR) splice variant 7 and full-length AR expression is associated with clinical outcome: a translational study in patients with castrate-resistant prostate cancer. BJU Int.

[219] Joncas F-H, Lucien F, Rouleau M, Morin F, Leong HS, Pouliot F (2019). Plasma extracellular vesicles as phenotypic biomarkers in prostate cancer patients. Prostate.

[220] Fujita K, Nonomura N (2019). Role of androgen receptor in prostate cancer: a review. World J Mens Health.

[221] Del Re M, Biasco E, Crucitta S, Derosa L, Rofi E, Orlandini C (2017). The detection of androgen receptor splice variant 7 in plasma-derived exosomal RNA strongly predicts resistance to hormonal therapy in metastatic prostate cancer patients. Eur Urol.

[222] Nimir M, Ma Y, Jeffreys SA, Opperman T, Young F, Khan T (2019). Detection of AR-V7 in liquid biopsies of castrate resistant prostate cancer patients: a comparison of AR-V7 analysis in circulating tumor cells, circulating tumor RNA and exosomes. Cells..

[223] Woo H-K, Park J, Ku JY, Lee CH, Sunkara V, Ha HK (2019). Urine-based liquid biopsy: non-invasive and sensitive AR-V7 detection in urinary EVs from patients with prostate cancer. Lab Chip.

[224] Yu Q, Li P, Weng M, Wu S, Zhang Y, Chen X (2018). Nano-vesicles are a potential tool to monitor therapeutic efficacy of carbon ion radiotherapy in prostate cancer. J Biomed Nanotechnol.

[225] Malla B, Aebersold DM, Dal Pra A (2018). Protocol for serum exosomal miRNAs analysis in prostate cancer patients treated with radiotherapy. J Transl Med.

[226] Kato T, Mizutani K, Kawakami K, Fujita Y, Ehara H, Ito M (2020). CD44v8-10 mRNA contained in serum exosomes as a diagnostic marker for docetaxel resistance in prostate cancer patients. Heliyon..

[227] Ishizuya Y, Uemura M, Narumi R, Tomiyama E, Koh Y, Matsushita M (2020). The role of actinin-4 (ACTN4) in exosomes as a potential novel therapeutic target in castration-resistant prostate cancer. Biochem Biophys Res Commun.

[228] Bhagirath D, Yang TL, Tabatabai ZL, Majid S, Dahiya R, Tanaka Y (2019). BRN4 is a novel driver of neuroendocrine differentiation in castration-resistant prostate cancer and is selectively released in extracellular vesicles with BRN2. Clin Cancer Res.

[229] Thompson TC, Timme TL, Li L, Goltsov A (1999). Caveolin-1, a metastasis-related gene that promotes cell survival in prostate cancer. Apoptosis..

[230] Thompson TC, Tahir SA, Li L, Watanabe M, Naruishi K, Yang G (2010). The role of caveolin-1 in prostate cancer: clinical implications. Prostate Cancer Prostatic Dis.

[231] Tahir SA, Ren C, Timme TL, Gdor Y, Hoogeveen R, Morrisett JD (2003). Development of an immunoassay for serum caveolin-1: a novel biomarker for prostate cancer. Clin Cancer Res..

[232] Tahir SA, Frolov A, Hayes TG, Mims MP, Miles BJ, Lerner SP (2006). Preoperative serum caveolin-1 as a prognostic marker for recurrence in a radical prostatectomy cohort. Clin Cancer Res.

[233] Ayers L, Pink R, Carter DRF, Nieuwland R (2019). Clinical requirements for extracellular vesicle assays. J Extracell Vesicles.

[234] Moller, A, Lobb, RJ. The evolving translational potential of small extracellular vesicles in cancer. Nat Rev Cancer. 2020;20:697–709. 10.1038/s41568-020-00299-w 32958932

[235] Yekula A, Muralidharan K, Kang KM, Wang L, Balaj L, Carter BS (2020). From laboratory to clinic: translation of extracellular vesicle based cancer biomarkers. Methods.

[236] Consortium E-T, Van Deun J, Mestdagh P, Agostinis P, Akay O, Anand S (2017). EV-TRACK: transparent reporting and centralizing knowledge in extracellular vesicle research. Nat Methods.

[237] Zhang H, Freitas D, Kim HS, Fabijanic K, Li Z, Chen H (2018). Identification of distinct nanoparticles and subsets of extracellular vesicles by asymmetric flow field-flow fractionation. Nat Cell Biol..

[238] Lasser C, Shelke GV, Yeri A, Kim DK, Crescitelli R, Raimondo S (2017). Two distinct extracellular RNA signatures released by a single cell type identified by microarray and next-generation sequencing. RNA Biol.

[239] Lunavat TR, Cheng L, Kim DK, Bhadury J, Jang SC, Lasser C (2015). Small RNA deep sequencing discriminates subsets of extracellular vesicles released by melanoma cells—evidence of unique microRNA cargos. RNA Biol.

[240] Pavlou MP, Diamandis EP, Blasutig IM (2013). The long journey of cancer biomarkers from the bench to the clinic. Clin Chem.

[241] Pepe MS, Etzioni R, Feng Z, Potter JD, Thompson ML, Thornquist M (2001). Phases of biomarker development for early detection of cancer. J Natl Cancer Inst.

[242] Fuzery AK, Levin J, Chan MM, Chan DW (2013). Translation of proteomic biomarkers into FDA approved cancer diagnostics: issues and challenges. Clin Proteom..

